# Hologenome analysis reveals independent evolution to chemosymbiosis by deep-sea bivalves

**DOI:** 10.1186/s12915-023-01551-z

**Published:** 2023-03-08

**Authors:** Yang Guo, Lingfeng Meng, Minxiao Wang, Zhaoshan Zhong, Denghui Li, Yaolei Zhang, Hanbo Li, Huan Zhang, Inge Seim, Yuli Li, Aijun Jiang, Qianyue Ji, Xiaoshan Su, Jianwei Chen, Guangyi Fan, Chaolun Li, Shanshan Liu

**Affiliations:** 1 Center of Deep-Sea Research, Institute of Oceanology, Chinese Academy of Sciences, Qingdao, 266071 China; 2 BGI-Qingdao, BGI-Shenzhen, Qingdao, 266555 China; 3 College of Life Sciences, University of Chinese Academy of Sciences, Beijing, 100049 China; 4 Pilot National Laboratory for Marine Science and Technology, Qingdao, 266237 China; 5 BGI-Shenzhen, Shenzhen, 518083 China; 6 Integrative Biology Laboratory, College of Life Sciences, Nanjing Normal University, Nanjing, 210046 China; 7 School of Biology and Environmental Science, Queensland University of Technology, Brisbane, QLD 4000 Australia; 8 College of Marine Life Sciences, Ocean University of China, Qingdao, 266003 China; 9 College of Marine Science, University of Chinese Academy of Sciences, Qingdao, 266400 China; 10 South China Sea Institute of Oceanology, Chinese Academy of Sciences, Guangzhou, 510301 China; 11 Qingdao Key Laboratory of Marine Genomics, BGI-qingdao, Qingdao, China

**Keywords:** Symbiosis, Hydrothermal vent, Bivalvia, *Conchocele bisecta*, Genome, Stochastic evolution, Comparative genomics, Phagocytosis, Lipopolysaccharide scavenging

## Abstract

**Background:**

Bivalves have independently evolved a variety of symbiotic relationships with chemosynthetic bacteria. These relationships range from endo- to extracellular interactions, making them ideal for studies on symbiosis-related evolution. It is still unclear whether there are universal patterns to symbiosis across bivalves. Here, we investigate the hologenome of an extracellular symbiotic thyasirid clam that represents the early stages of symbiosis evolution.

**Results:**

We present a hologenome of *Conchocele bisecta* (Bivalvia: Thyasiridae) collected from deep-sea hydrothermal vents with extracellular symbionts, along with related ultrastructural evidence and expression data. Based on ultrastructural and sequencing evidence, only one dominant Thioglobaceae bacteria was densely aggregated in the large bacterial chambers of *C. bisecta*, and the bacterial genome shows nutritional complementarity and immune interactions with the host. Overall, gene family expansions may contribute to the symbiosis-related phenotypic variations in different bivalves. For instance, convergent expansions of gaseous substrate transport families in the endosymbiotic bivalves are absent in *C. bisecta*. Compared to endosymbiotic relatives, the thyasirid genome exhibits large-scale expansion in phagocytosis, which may facilitate symbiont digestion and account for extracellular symbiotic phenotypes. We also reveal that distinct immune system evolution, including expansion in lipopolysaccharide scavenging and contraction of IAP (inhibitor of apoptosis protein), may contribute to the different manners of bacterial virulence resistance in *C. bisecta*.

**Conclusions:**

Thus, bivalves employ different pathways to adapt to the long-term co-existence with their bacterial symbionts, further highlighting the contribution of stochastic evolution to the independent gain of a symbiotic lifestyle in the lineage.

**Supplementary Information:**

The online version contains supplementary material available at 10.1186/s12915-023-01551-z.

## Background

Since the beginning of metazoan evolution, the development of animals has depended on the interactions with bacterial communities [1–3], and symbiotic relationships between microbial partners and animal hosts are hallmarks of these associations [4, 5]. Symbiosis has evolved independently in each lineage and is frequently associated with evolutionary adaptations [6, 7]. Thus, symbiosis-related evolution is a substantial contributor to phenotypic complexity and has long been a hot topic in biology [8–11].

Deep-sea chemosynthetic ecosystems are patchily distributed and turbulent [12]. Chemosynthetic bacteria are the primary producers of deep-sea cold seeps and vents [7, 13]. Thus, symbiosis is a common phenomenon in this ecosystem to facilitate energy utilization. As one representative of deep-sea symbiotic organisms, the widely distributed bivalves have shown variations in symbiosis-related traits, such as the spatial structure of the holobionts and intimacy with the symbionts, which make them ideal materials for studies on the correlations between symbiosis and evolution [14, 15].

According to Wein et al., the divergences of symbioses are determined by three elements: symbiotic currency, mechanism of currency exchange, and inheritance regimes [16]. Among them, symbiotic currency is constant among chemosymbiotic bivalves, where the host provides steady inorganic substrates to symbionts for carbon fixation, and in return, the bivalves obtain fixed carbons and rare metabolites, for example, the essential amino acids for bivalves [14, 17, 18]. However, the other two elements in deep-sea Bivalvia symbiotic systems have been shown to be highly varied.

The currency exchange mechanism varies and depends on the spatial structure of the holobionts. Most symbiotic bivalves belonging to Vesicomyinae and Bathymodiolinae host their symbionts intracellularly while most symbiotic thyasirids host their bacterial symbionts extracellularly [19, 20]. Correspondingly, the nutrient transfer mode may differ among taxa. Endosymbiotic bivalves, such as *Archivesica marissinica*, import gaseous substrates using carbonic anhydrase and globin so that intracellularly located symbionts can drive carbon fixation [21, 22]. In extracellular symbiotic thyasirids, the bacterial symbionts are located outside the cell membrane of bacteriocytes, which would allow the symbionts to obtain substances directly from the environment [20]. Moreover, many symbionts are alive in the bacteriocytes of endosymbiotic mussels [23]. In contrast, the extracellular symbionts of thyasirids are endocytosed in phagosomes, and the digestion rate of bacteria in phagosomes is much higher in the bacteriocytes [20, 24]. Thus, with the phenotypic divergence in spatial structure and currency exchange, how thyasirids differ from the endosymbiotic bivalves in the genome remains debated.

The inheritance regimes of bivalves can be divided into vertical inheritance from parents to offspring and horizontal transmission from the environment [25]. How the hosts recognize and police the bacterial symbionts are crucial in shaping these inheritance regimes. Different patterns in bacterial recognition and immune response have been demonstrated by comparative genomic investigations of endosymbiotic bivalves. For example, the mussel *Gigantidas platifrons* manifests horizontal transmission and expansion of gene families involved in immunological recognition [26]. The same families are contracted in the clam *A. marissinica*, which manifests vertically transmitted symbiosis [22]. The extracellularly symbiotic thyasirids might have evolved a distinct mechanism in bacterial recognition and homeostasis since they are considered less integrated with their symbionts [27]. First, they harbor bacteria either among the microvilli outside the epithelial cells of gills or, more integrally, in the apical vesicles delimited by the cell membrane and microvilli (hereafter for extracellular symbiosis to distinguish from the previous type of epi-symbiosis) [20]. In addition, thyasirids show a fluctuating dependence upon symbiont-derived nutrients [28, 29]. Thus, extracellular symbiotic thyasirids might be regarded as intermediate between endosymbiotic and asymbiotic bivalves and may exhibit distinct evolutionary adaptations. In addition, how the thyasirid hosts select and recruit their symbionts with high specificity is still unknown.

Here, we present a genome of *Conchocele bisecta* (Bivalvia: Thyasiridae) and its extracellular symbiont. By comparative genomic analysis of *C. bisecta* and endosymbiotic bivalves, genetic divergence related to the variations in currency exchange and bacterial recognition and policing are revealed. Ultrastructural observations and gene expression further corroborated our findings. We tested the hypothesis that the deep-sea bivalves evolve independently to give rise to the convergent symbiosis characteristics. Our results highlight that stochastic evolution may shape divergent symbiosis interactions by deep-sea bivalves.

## Results and discussion

### Distribution and constitution of symbionts in *C. bisecta*

**Fig. 1:**
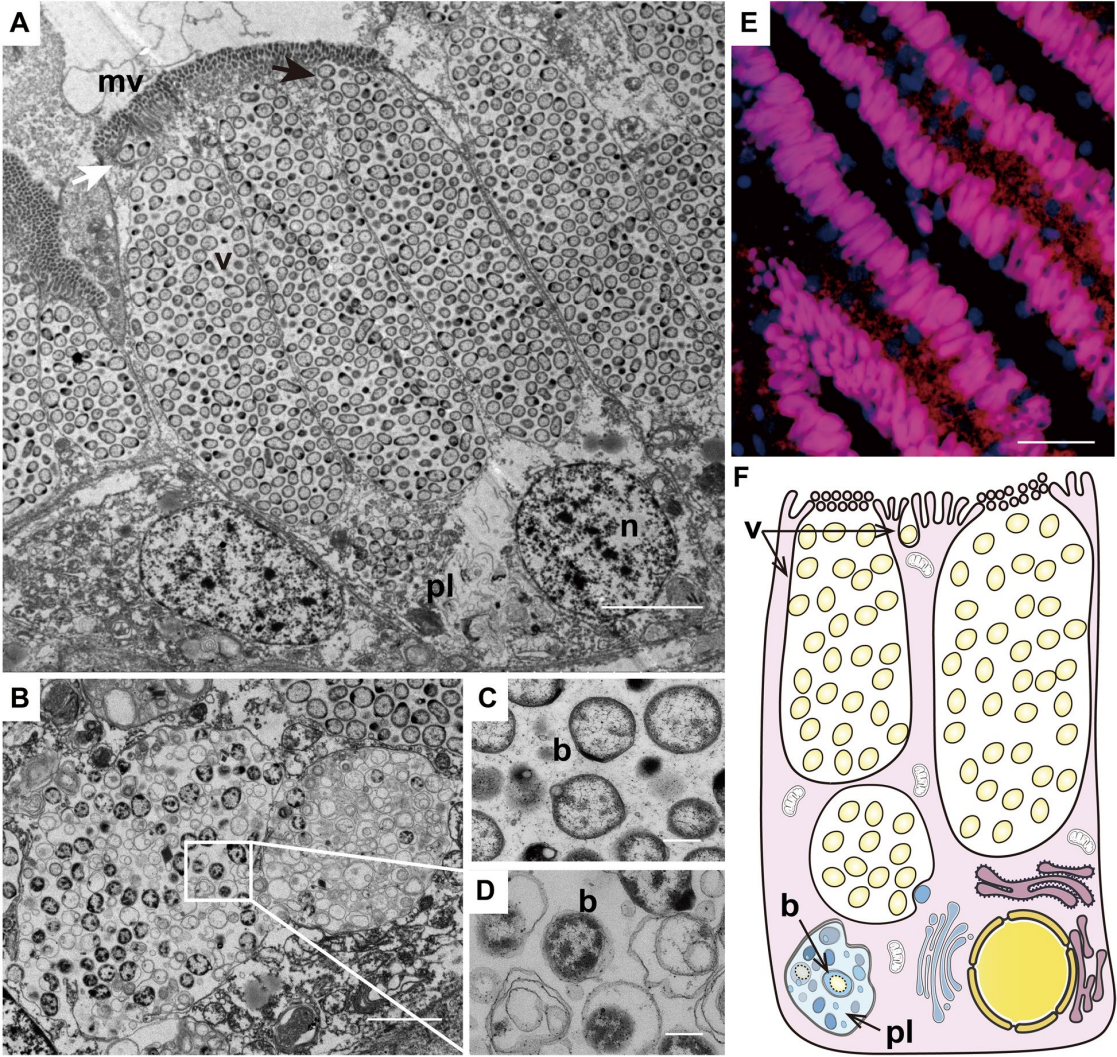
Distribution of bacterial symbionts in the gill filaments of *Conchocele bisecta* collected in a deep-sea hydrothermal vent field*. ***A** Transmission electron micrography (TEM) of bacteriocytes in gill filaments. Extracellularly symbionts were densely aggregated in the large apical vesicles (black arrow; v: vesicle) covered by microvilli (mv) and smaller vesicles (white arrow). Phagolysosome-like organelles (pl) and nucleus (n) located in the basolateral membranes of the bacteriocytes (scale bar: 5 μm). **B** Details of phagolysosome-like organelles with whorls of lysed bacterial products (scale bar 2 μm). **C** Living bacterial symbiont (b) with a clear border located in the apical vesicles, and the symbionts were small cocci with a diameter around 500 nm (scale bar 250 nm). **D** Lysed bacterial symbionts with blurred cell walls in the phagolysosome-like organelles (scale bar: 250 nm). **E** Fluorescence in situ hybridization (FISH) of 16S rRNA of the dominate symbionts in the gill filaments. The image shows the overlay of DAPI-stained DNAs and 16S rRNA tagged with Cy3 dye (scale bar: 50 μm). **F** Schematic representation of typical bacteriocytes, including small, and large apical vesicles (v), phagolysosome-like organelles (pl), and lysed symbionts (b)

To determine how bacterial symbionts are distributed in *C. bisecta* sampled from hydrothermal vents (Additional file 1: Supplementary note 1, Fig. S1) [30, 31], transmission electron micrography (TEM) was performed. Densely aggregated bacterial symbionts were in apical vesicles of gill filaments bacteriocytes (Fig. 1A). In most cases, the apical vesicles were opened at the tip but covered by microvilli, while lysosomes and other organelles located near the basal membrane side (Fig. 1A, B). In addition to long, large columnar vesicles, we noticed that there were also small ones, which were likely to be at an initial stage (Fig. 1A). In contrast to the bacterial symbionts in the apical vesicles (Fig. 1C), symbionts in intracellular vesicles had blurred cell walls and irregular shapes (Fig. 1D). It has been reported that thyasirids obtain nutrients by periodically engulfing and digesting their symbionts [24, 32]. These results suggest that most symbionts are quickly digested after being endocytosed by bacteriocytes of *C. bisecta*, and most of these intracellular vesicles should be organelles such as late phagosomes and phagolysosomes. In the endosymbiotic deep-sea mussels and vesicomyid clams, the phenotype is quite different: despite the presence of vesicles that appeared to be digesting the symbionts, there were many vesicles holding live endosymbionts, indicating that many of these vesicles might be early phagosomes or at more primitive stages [33, 34].

Based on the 16S rDNA V4 region amplicon analysis, bacteria close to the clade SUP05 dominated in the gill tissue of *C. bisecta* with a relative abundance of 94.73±3.20% (Additional file 2: Table. S1). Fluorescence in situ hybridization (FISH) was conducted to verify this observation. Probes (16S-Probe) were designed based on 16S rDNA sequences of the dominated symbionts, and a strong signal was detected in columnar vesicles arranged in the gill filaments, in accordance with the TEM examination. The negative control probe did not hybridize with both tissues, and no positive signal was observed in gonad tissue (Fig. 1E, Additional file 1: Fig. S2). The bacteria-host specificity has been widely reported in multiple lineages, including corals, nematodes, and mollusks [35–37]. Although the mechanism underlying this phenomenon is still being debated, the vertical transmission of symbionts and immune interactions were thought to be significant contributors [36–38]. However, according to our data, the dominant symbionts were absent in the gonad tissue of the *C. bisecta*, supporting the theory that thyasirids acquire symbionts horizontally [39, 40]. Thus, the genomic characterization of the holobionts would be necessary to provide insights into the interactions and specificities between host and its symbionts.

A high-quality genome of the extracellular bacterial symbiont of *C. bisecta* (hereafter SCbi) with a 92.85% completeness was recovered through a binning pipeline. The assembly consists of three single-copy rRNAs and 31 tRNAs (Additional file 1: Table S2). The 16S rDNA sequences and the GTDB (Genome Taxonomy Database) taxonomy annotation agreed to assign SCbi as *Thiodubiliella* sp. Interestingly, we found some genomic features that may contribute to symbiotic life by providing fixed carbons and rare metabolites to the thyasirid host. In brief, SCbi uses a modified Calvin cycle for carbon fixation driven by sulfur oxidation. We identified many copies of SulP-type transporters in the symbiont genome (Additional file 2: Table S3) [41, 42]. Members of the SulP family are associated with the intake of inorganic anions, such as sulfates and bicarbonates, in prokaryotes [43, 44], suggesting that these genes facilitate chemoautotrophy. The SCbi genome exhibited complete routes for biosynthesis of 18 of 20 amino acids and five of ten cofactors (Additional file 1: Fig. S3, S4, Additional file 2: Table S4). As supported by proteomic data, missing enzymes in the biosynthesis of the amino acid threonine and folate may be compensated by a different enzyme or reaction products provided by the host (Additional file 1: Supplementary note 2, Fig. S3–7 and Table S5, Additional file 2: Table S4) [22, 45, 46]. Additionally, genes related to lipopolysaccharide (LPS) modifications and pathogenic invasion, such as homologs of *slyB* and *yeeJ*, were found in the SCbi genome and may be implicated in symbiotic interactions. For instance, according to our proteomic data, the invasin, encoded by *yeeJ* (intensity-based absolute quantification (iBAQ) per million: 512.64), and the host receptor of integrin-beta1 (iBAQ per million: 49.18) were both actively translated in the gill tissue, pointing to a potential pathway of bacteria triggered phagocytosis or invasion [47, 48].

Taken together, *C. bisecta* hosts bacterial symbionts in large apical vesicles of bacteriocytes in gill tissue, similar to the previously reported cold seep *C. bisecta*, and other thyasirids such as *Thyasirid flexuosa* [20, 49]. Moreover, *C. bisecta* harbors a dominant *Thiodubiliella* symbiont that appears adequate for providing most necessary rare metabolites to the host and interacting immunologically with the host. Moreover, our TEM results emphasize differences between the intracellularly symbiont-holding vesicles of the extracellular symbiotic *C. bisecta* and endosymbiotic mussels and clams. The absence of SCbi in gonad tissue further supports that *C. bisecta* acquired its bacterial symbiont from the surrounding environments horizontally, highlighting the importance of bacterial recognition in this symbiotic relationship.

### Host genome assembly and phylogenetic analysis

**Fig. 2:**
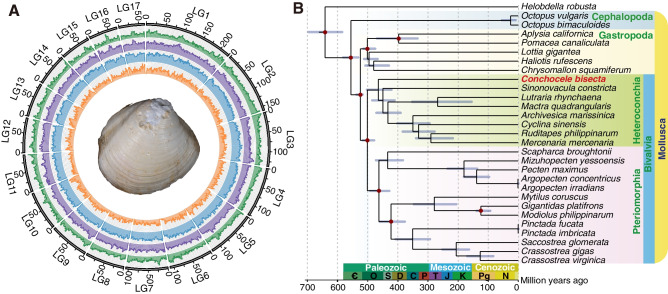
Genomic characteristics of *Conchocele bisecta*. **A** Circos view of 17 linkage groups (corresponding to “pseudo-chromosomes”) showing marker distributions at 2-Mb sliding windows from outer to inner circle: GC content, TE density, tandem repeat density, gene density. **B** Maximum-likelihood phylogenetic relationships were constructed using the LG+F+R6 substitution model among 28 mollusks with an annelid (*Helobdella robusta*) as an outgroup. The tree was calibrated at nine nodes (indicated by red dots) using divergence times from TimeTree (http://www.timetree.org). Blue lines indicate 95% confidence intervals for divergence times

To decipher the genetic machinery under these unique symbiotic features, we assembled a high-quality genome of *C. bisecta* by combining long-read PacBio sequencing reads with highly accurate short reads generated on the BGISEQ 500 platform and used Hi-C data for super-scaffolding (Additional file 1: Supplementary Note 3, Fig. S6 and Table S6–9). In detail, the 1.9 Gb assembly contained 17,791 contigs with a contig N50 length of 488 kb (Fig. 2A, Additional file 1: Table S7). We annotated 25,483 protein-coding genes and recovered 95.4% of metazoan BUSCOs (Benchmarking Universal Single Copy Orthologs) (Additional file 1: Table S7).

Our phylogenetic results showed that *C. bisecta* from Lucinida lies in a basal position of the heterodont clade, and the divergence time between *C. bisecta* and other heterodont clams is estimated as 464.9 million years ago (mya) in the mid-Ordovician (Fig. 2B). As recently reported, the oldest fossil record of thyasirid bivalve is *Eothyasira antiqua* from the early Jurassic [50]. However, it was previously suspected, employing 18S rRNA gene data, that Thyasiridae have a longer fossil history than its published records [51], and our phylogenetic results support this long divergence time. The conservation index (CI) of six bivalves with ancestral linkage groups (ALG) that are represented by genes of *Nematostella vectensis* were calculated by a chromosome-based macrosynteny analysis [52]. Although the CI of *C. bisecta* (0.73) is not the highest among heterodont bivalves, the CIs of heterodont bivalves fell in a narrow window (0.7–0.8), which was much higher than that of the pearl oyster (0.45) (Additional file 1: Fig. S9). The accordant high CIs of heterodont bivalves indicated the genome organization of this clade is highly conserved to bilaterian ancestral genomes, although with a long divergence time among these clams.

Integrating repeat content results from hybrid software revealed that the transposable element (TE) content of *C. bisecta* is strikingly high. About 6.5 million repetitive elements were identified, accounting for the relatively large genome of the species (Additional file 1: Fig. S10a). Comparative analysis further revealed that the genome of *C. bisecta* contains both the largest amount (1.27 Gb) and the highest percentage (66.96%) of TEs than genomes of other lineages (Additional file 1: Fig. S10a and Table S10). It has been argued that TE content contributes to larger genome sizes [53–55]. We found that the genome size and TE content correlated in 27 lophotrochozoan genomes, except for the symbiotic clams *C. bisecta* and *A. marissinica*, which showed an elevated proportion of TEs (Additional file 1: Fig. S10b). Moreover, the two symbiotic clams had longer introns than other heterodont clams, which could be the results of high TE contents in the genomes, as seen in the African lungfish (Additional file 1: Fig. S10c) [54]. Expansions in TE-related protein domains have been shown to contribute to the high TE proportions in *A. marissinica* [22]. These protein domains, including the reverse transcriptase domain, DDE superfamily endonuclease, and the MULE transposase domain, were also significantly expanded in the *C. bisecta* genome (Additional file 1: Fig. S11). TEs can replicate independently within their host genomes and are a major source of genetic variation and novelty [56]. Thus, based on our data, the expansion in TE-related protein domains and the large amounts of TEs in these symbiotic clams may provide sufficient evolutionary material for the hosts to adapt to the symbiotic lifestyle, or at the very least, to diverse environments.

### Evolution of gene families and their roles in symbiosis

Bivalves are ideal materials for studies on symbiosis and evolution due to the diverse symbiotic forms among different lineages [14, 15]. To the best of our knowledge, only two genomes of symbiotic bivalves, *A. marissinica* and *G. platifrons*, have been published [22, 26]. As described, both species were endosymbiotic, with *A. marissinica* appearing to be more integrated with its symbionts than *G. platifrons* did. To investigate the evolution of symbiosis by bivalves and compare the genetic machinery under different symbiosis-related traits, we compared extracellular symbiotic *C. bisecta* and endosymbiotic *A. marissinica* and *G. platifrons* with five asymbiotic bivalves (*Crassostrea gigas*, *Lutraria rhynchaena*, *Mizuhopecten yessoensis*, *Modiolus philippinarum*, *Pinctada fucata*). The expansion and contraction events were identified both at gene and domain levels (Additional file 1: Fig. S11, Additional file 2: Table S11).

**Fig. 3:**
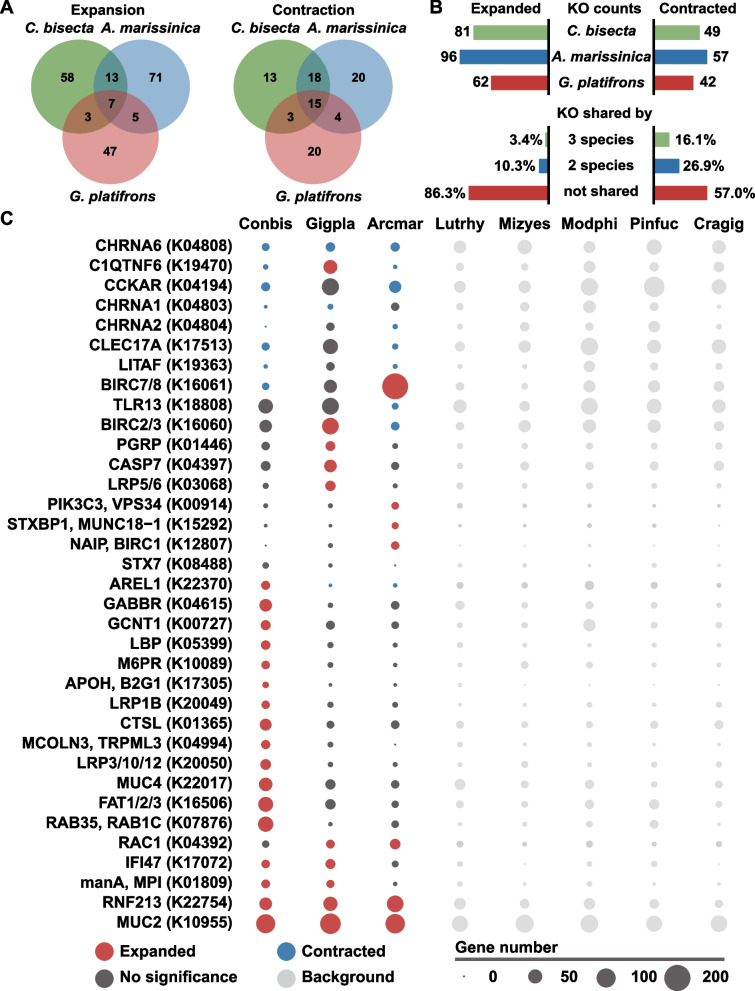
Gene family dynamics in the genomes of *Conchocele bisecta*, *Archivesica marissinica*, and *Gigantidas platifrons*. **A** Venn diagram showing the number of shared and unique expanded and contracted gene families in the three symbiotic species compared to five asymbiotic bivalves. Annotated to KEGG orthologs (KOs). **B** Total counts of KOs that expanded or contracted in each of the three bivalves, and the rates (%) of shared KOs. There are more exclusively expanded KOs than contracted ones in each bivalve. **C** Bubble plot of key expanded or contracted KOs in the three bivalves. Bubble size indicates the gene number of each KO in the eight species: *C. bisecta* (Conbis), *G. platifrons* (Gigpla), *A. marissinica* (Arcmar), *Lutraria rhynchaena* (Lutrhy), *Mizuhopecten yessoensis* (Mizyes), *Modiolus philippinarum* (Modphi), *Pinctada fucata* (Pinfuc), *Crassostrea gigas* (Cragig). Significant expansion (red), contraction (blue), or no significance (dark gray) were determined by Fisher’s exact test (*P* < 0.05)

For each of the three symbiotic bivalves, there appeared to be fewer contracted KEGG orthologs (KOs) than expanded KOs, but nearly half of these contracted KOs were shared by the bivalves (Fig. 3A, B). Considering the independent origin of the taxa compared, the shared contracted KOs might be related to the convergent adaptations, such as adaptions to nutritional symbiosis in the deep sea. For instance, the cholecystokinin receptor (CCKAR) with roles in bivalve digestion was contracted in *C. bisecta* and *A. marissinica*, in agreement with their reduced digestive systems (Fig. 3C, Additional file 2: Table S11) [57, 58]. In addition, among the contracted KOs, several reduced acetylcholine receptors (CHRNAs) gene families were shared by all three symbiotic species (Fig. 3C, Additional file 2: Table S11). The adaptation to a lifestyle of coexisting with bacterial symbionts might be related to these contraction events since CHRNAs are implicated in regulating immune response and pathogen removal in both vertebrates and invertebrates (including bivalves) [59, 60].

We found many gene families expanded specifically in different lineages. Hence, we propose that the uniquely evolved KOs, especially the large amounts of expanded KOs, might be related to the variations of symbiosis-related phenotypes among the three bivalves, such as the spatial structures and the ways of symbiont inheritance. In agreement with this hypothesis, KOs related to the divergent symbiotic traits in bivalves (e.g., nutrient transport, phagocytosis, bacterial recognition, and immune response) were included in each list of the expanded KOs (Fig. 3C, Additional file 2: Table S11). This indicates that gene family expansions may contribute to the adaptive evolution of symbiosis and in shaping the different symbiotic phenotypes in *C. bisecta* and other bivalves. Thus, we further investigated the expanded KOs.

### Symbionts as the primary nutrient supply for the host

**Fig. 4:**
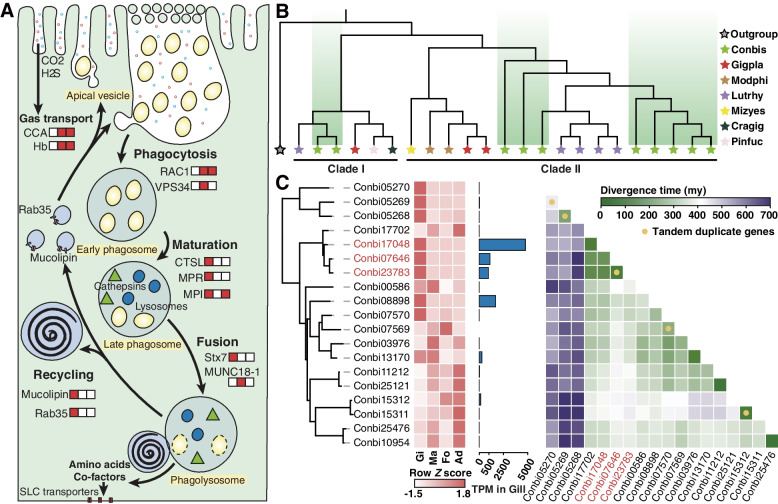
Gene families related to symbiotic currency exchange in *Conchocele bisecta*. **A** Schematic representation of currency exchange in *C. bisecta*. Expansion events of gene families responsible for substrate transports and phagocytosis are illustrated, and the boxes that followed each gene indicate if the gene family is expanded (red) or not (white) in *C. bisecta* (right), *Archivesica marissinica* (middle), and *Gigantidas platifrons* (left). **B** Phylogenetic tree based on amino acids of mucolipins (Conbis: *C. bisecta*; Gigpla: *G. platifrons*; Lutrhy: *Lutraria rhynchaena*; Mizyes: *Mizuhopecten yessoensis*; Modphi: *Modiolus philippinarum*; Pinfuc: *Pinctada fucata*; Cragig: *Crassostrea gigas*; Outgroup: *Danio rerio*). **C** Phylogenetic relationships of cathepsin L genes (*ctsL*) in *C. bisecta*. Expression levels of different tissues (Gi, gill; Ma, mantle; Fo, foot; Ad, adductor) and Row-wise *Z*-score of TPM (transcript per million) counts in gill tissue and gene divergence times (My, million years) are shown if *ctsL* copy lengths range from 600 to 1200 bp, and yellow dots indicate the tandem duplicate gene pairs. Three duplicated genes (in red) with short divergent time constitute most of the *ctsL* transcripts in gill tissue

With the absence of biosynthesis capabilities of some amino acids and cofactors, it is important for mollusks to derive nutrients from external sources or their bacterial symbionts [61]. *C. bisecta* was unable to synthesize eight amino acids, including histidine, phenylalanine, isoleucine, lysine, leucine, valine, tryptophan and methionine, and genes involved in the uptake these amino acids, such as *Slc6a19*, *Slc7a9*, and *Slc16a10*, were found in the genome (Additional file 1: Fig S3, S12, Additional file 2: Table S4). SLC16A10, a transporter mediating the diffusion of amino acids across basolateral membranes of the epithelial cells [62], is both expanded and highly expressed in the gills of *C. bisecta* (Additional file 1: Fig. S12). As described, *C. bisecta* digests its symbiont intracellularly, and amino acids released by the symbionts should be accumulated in bacteriocytes, which is in correspondence to the actively transcribed *Slc16a10* (Fig. 4A). Similarly, transporters for vitamin B5 (VB5), VB7, and VB12 are highly expressed in the symbiotic tissue of *C. bisecta* (Additional file 1: Supplementary note 4, Fig. S4, S12, Additional file 2: Table S4). Therefore, the highly expressed gene family of these transporters in *C. bisecta* could emphasize the nutritional function of its gill tissues. Moreover, gene families coding for enzymes related to the filter-feeding lifestyle in mollusks, such as the glycosyl hydrolase and chymotrypsins, are found either contracted or missing in *C. bisecta*, suggesting that the clam had relied on symbiosis for nutrition for a long time (Additional file 1: Supplementary note 5, Additional file 2: Table S12) [22, 28, 63–65].

Unlike bivalves with endosymbionts, we did not find an expansion of gene families required for gaseous substance transport in *C. bisecta* (Additional file 1: Fig. S13, Fig. S14). Bacterial symbionts are located outside the cell of *C. bisecta*, and the symbionts could acquire gaseous substrates from the seawater directly (Fig. 4A). Thus, it is reasonable that there is no expansion of these transporters. As the endosymbionts obtain the gaseous substances from the cytoplasm, cytoplasmic carbonic anhydrase (CCA) and hemoglobin (Hb) that may be involved in the transportation of CO_2_ and sulfide and oxygen for carbon fixation are expanded in their hosts [22, 65, 66]. These findings support the hypothesis that an expansion of CCAs and Hbs in endosymbiotic bivalves is adaptive [66].

To summarize, evolutionary divergences in gene families associated with gaseous substrate transport might depend on whether symbionts are located inside bacteriocytes. In addition, the high expression of transporters of rare metabolites in symbiotic tissues and the contraction in filter-feeding-related genes revealed a high reliance on nutritional symbiosis by *C. bisecta*, and the results underlined the convergences in symbiotic currencies among the chemosymbiotic bivalves.

### Enhanced phagocytic capabilities provide insights into extracellular symbiosis

As revealed by previous studies and our TEM examination (Fig. 1), phagocytosis was considered to contribute significantly to nutritional currency exchange in extracellular symbiotic thyasirids and two endosymbiotic bivalves [20, 24, 33, 34]. Accordingly, phagocytosis-related gene families are expanded in all three symbiotic bivalves. However, the evolutionary pattern of these genes in *C. bisecta* is distinct from the endosymbiotic ones (Fig. 4A).

Phagocytosis in eukaryote cells includes bacterial internalization, digestion, and the recycling of lysosomes. Remarkably, the *C. bisecta* genome exhibit a series of expansion events that related to the processes of lysosome-mediated intracellular digestion and lysosome recycling, including the mannose-6-phosphate receptor (MPR), cathepsin L (CSTL), Syntaxin7 (Stx7), mucolipin (MCOLN), and Ras-related protein Rab-35 (Rab35) (Fig. 4A, Additional file 2: Table S11). The above processes may be critical for the regulation and digestion of symbionts, and the related genes are highly expressed in symbiotic tissues [67, 68]. However, according to our analysis, they are only significantly expanded in *C. bisecta*, highlighting their possible contribution on extracellular symbiosis. For instance, MPR plays a key role in the phagosome maturation by importing hydrolases to phagosome [69]. In contrast to 2 or 5 copies coded by the endosymbiotic bivalves, 15 gene copies of MPR were found in *C. bisecta* genome (Fig. 3C), and four are specifically expressed in the gill tissues (Additional file 1: Fig. S15). Moreover, an expanded set of MCOLN genes, which are crucial for lysosomes recycling [70–72], is present in *C. bisecta*. Based on phylogenetic relationships, MCOLNs split into two clades in bivalves, and thyasirid MCOLNs expanded in both clades and clustered with their heterodont relatives (Fig. 4B). However, based on our analysis, genes implicated in the internalization of the symbionts were only expanded in the endosymbiotic bivalves but the extracellular symbiotic *C. bisecta* (Fig 4A). For example, Rac family small GTPase 1 (Rac1) and phosphatidylinositol 3-kinase (VPS34) were key factors in this process [73, 74]. These two families were only found to be expanded in the endosymbiotic bivalves, but not in the extracellular symbiotic *C. bisecta* (Fig. 3C, Additional file 2: Table S11). Thus, although phagocytosis-related expansion events were observed in all symbiotic bivalves, only the thyasirid genome exhibited overall expansion of bacterial digestion and subsequent lysosome recycling genes. In contrast, the endosymbiotic bivalves likely show enhanced bacterial internalization.

Interestingly, we found some genetic evidence related to a rapid bacterial clearance rate in *C. bisecta.* Compared to the endosymbiotic bivalves, cathepsins, the hydrolases responsible for the catabolic ability of lysosomes with high expression in gills, were significantly expanded in thyasirid (Fig. 3C, Additional file 1: Fig. S15) [22, 65, 66, 75]. Based on the divergence time of *ctsL* genes, four copies (Conbi17702, Conbi17048, Conbi07646 and Conbi23783) were estimated to have expanded in a narrow window, while two of them were identified as tandem duplicate pairs (Fig. 4C). Interestingly, among the four *ctsL* copies, three genes were exclusively expressed and accounted for most of the *ctsL* transcripts in gill tissue (Fig. 4C). We speculate the expansion events enhance bacterial digestion. The processes from early phagosome through symbiont digestion may be particularly efficient in the extracellular symbiotic *C. bisecta*, which corresponded to the phenotype of the thyasirids, where most internal vesicles retain lysed bacterial symbionts [24, 49, 76]. Furthermore, expanded gene families related to lysosome recycling have been implicated in forming a large phagocytic cup via promoting membrane extensions [77–79]. The large apical vesicles harboring densely aggregated *C. bisecta* symbionts were stunning. Additional membrane is required when the apical vesicles form, which might be related to these expansion events [72]. In addition, compared to endosymbiotic bivalves, the capability of internalizing symbionts might be relatively modest in *C. bisecta*, accentuating the above phenotypic variations between the extracellular symbiotic thyasirid and endosymbiotic bivalves.

In conclusion, the massive expansion in phagocytosis is in line with the theory that thyasirids periodically engulf and digest symbionts [28, 76]. In addition, the gene families related to phagocytosis divergently evolved among different bivalves. These divergences may be related to the phenotypic variations, such as the large apical vesicles in extracellular symbiotic clams, and the large amounts of endosymbiotic vesicles with live bacteria in both *A. marissinica* and *G. platifrons*.

### Immune system remodeling to the establishment and maintenance of extracellular symbiosis in *C. bisecta*

**Fig. 5:**
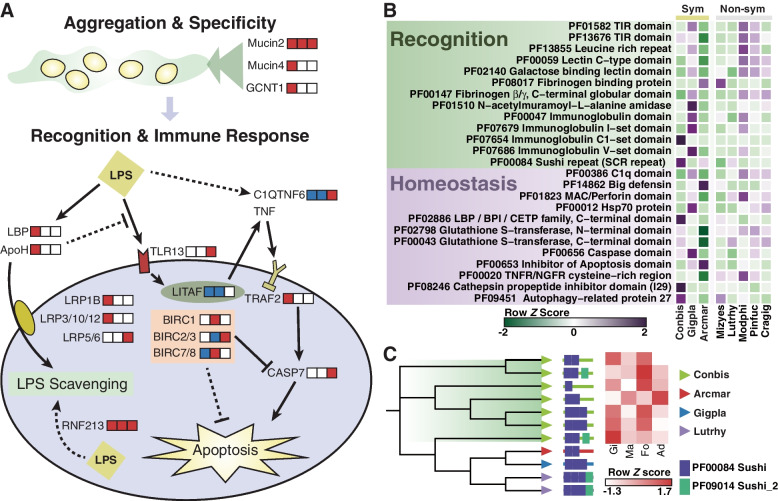
Gene family related to symbiosis establishment and maintenance in *Conchocele bisecta*. **A** Schematic representation of bacterial recognition and immune response in *C. bisecta*. Both expansion and contraction events of gene families involved in these processes are illustrated, and the boxes that followed each gene indicate if a gene family has expanded (red), contracted (blue), or neither (white) in *C. bisecta* (right), *Archivesica marissinica* (middle) and *Gigantidas platifrons* (left). **B** Heat maps of significantly evolved Pfam domains implicated in recognition and homeostasis in the three symbiotic (Sym) bivalves (Conbis: *C. bisecta*; Arcmar: *A. marissinica*; Gigpla: *G. platifrons*), compared with asymbiotic (non-sym) relatives (Lutrhy: *Lutraria rhynchaena*; Mizyes: *Mizuhopecten yessoensis*; Modphi: *Modiolus philippinarum*; Pinfuc: *Pinctada fucata*; Cragig: *Crassostrea gigas*). **C** Left, phylogenetic tree based on amino acids of apolipoprotein H. Predicted Pfam domains for each gene are shown. Right, heatmap showing the tissue-specific expression (Gi, gill; Ma, mantle; Fo, foot; Ad, adductor) of *ApoH* (row-wise *Z*-score of TPM counts)

According to our comparative genomic analysis, the immune system of *C. bisecta* is remodeled differently from that of *A. marissinica* and *G. platifrons* to make room for its extracellular symbiont (Fig. 5A). The roles of pattern recognition receptors (PRRs) in bacteria selection and capture may be reduced in thyasirid as four out of 13 PRR-related Pfam domains are contracted (Fig. 5B, Additional file 2: Table S13). However, the expansion of mucosal immunity-related genes, including mucin protein families and the GCNT1 (acetylglucosaminyltransferase) family for mucin glycosylation, has been observed, and these genes were functional in the gill tissues as are supported by both transcriptomes (Fig. 3C, Additional file 1: Fig. S16). As described, TEM examination and 16S rDNA analysis found SCbi was the only dominant bacteria in the apical vesicles of bacteriocytes, indicating that inter-partner recognition of symbionts has completed before the settlement of the symbionts in the apical vesicles. Thus, we speculate that the selection of symbionts occurred extracellularly possibly at the mucin layer based on both microscopic observation and comparative genomics. The mucosal interface is the first physical encounter where the interactions of microbe and host begin through bindings of host surface glycosylated proteins and symbiont sugars [25]. In the squid-vibrio system, it has been revealed that the glycosylated surface mucus secreted by the host is the key factor in recruiting and entraining of bacterial symbionts from seawater [5]. Therefore, although both *C. bisecta* and *G. platifrons* acquire symbionts by horizontal transmission, the expanded mucins and GCNT1 enzymes in *C. bisecta* are likely to function in bacterial symbiont aggregation and specificity of the bacterial symbionts, rather than via PRRs as in deep-sea mussels.

Once symbiotic relationships are established, the bacteriocyte may suffer from stressors like ROS (reactive oxygen species) due metabolic waste from symbionts. Hence, homeostasis maintenance strategies are needed to sustain the symbiosis system. Inhibitors of apoptosis protein (IAPs) are known to inhibit apoptosis and promote cell cycle progression and are expanded and highly expressed in gills of endosymbiotic bivalves to maintain cellular homeostasis (Fig. 5A) [22, 26]. However, the copy number of IAP Pfam domains in *C. bisecta* [37] is somewhat lower than the asymbiotic bivalves (56 on average) and much lower than endosymbiotic bivalves (232 for *A. marissinica*, 123 for *G. platifrons*) (Fig. 5A, B). In *C. bisecta*, most symbionts remain in apical vesicles among the microvilli of bacteriocytes and are digested once entering bacteriocytes. Thus, population control of symbionts by suppressing host cell apoptosis may not be required.

Although mainly located outside the cell, the thyasirids nevertheless need to accommodate the lifestyle of extracellular symbionts resident at their gills and foot. LPS is a common cell surface antigen of Gram-negative bacteria and one of the most potent initiators of inflammation and apoptosis, which can induce cytokine production and cause cytotoxic effects in animals, including mollusks (Fig. 5A) [80, 81]. We found that several gene families coding LPS-binding proteins expanded substantially and are highly expressed in both tissues of gill and foot (Fig. 5C). For example, apolipoprotein H (ApoH) can associate with and scavenge LPS by binding members of the low-density lipoprotein receptor-related protein (LRP) family to reduce endotoxin concentration [82, 83]. In addition to the expansion of ApoH, an expansion of three LRP families, including the LRP1B (14 vs. 3.7 gene copies in all other seven analyzed bivalves), LRP3/10/12 (26 vs. 4.9 gene copies), and LRP4 (34 vs. 21.7 gene copies), were observed in *C. bisecta* (Fig. 5A, Additional file 2: Table S11). LRP1B genes are the most highly expressed family among the expanded LRPs and are mainly expressed in the gills and mantle, while LRP3/10/12 genes are mainly expressed in the three muscle tissues (Additional file 1: Fig. S16). These results support a hypothesis that the host may maintain the relationship with the bacterial symbionts by neutralizing the defects of bacterial LPS. As additional evidence, other gene families involved in the immune response to LPS were significantly evolved, including the tumor necrosis factors (TNF), lipopolysaccharide induced TNF factors (LITAFs), and E3 ligases (Fig. 5A, Additional file 2: Table S11). For instance, we found that the E3 ubiquitin-protein ligase ring finger protein 213 (RNF213) has expanded in symbiotic bivalves. This family was recently reported to induce antibacterial autophagy by ubiquitylating LPS in cytosol [84].

Taken together, significant genetic differences have been observed in the two bivalves that obtain symbionts horizontally. In *C. bisecta*, glycosylated mucins, rather than the PRRs, may aid in symbiont integration, which is crucial for the horizontal transmission of symbionts in thyasirids [39, 40]. Although the convergent expansion of IAPs in endosymbiotic bivalves was not observed in *C. bisecta*, the expansion and high expression of multiple gene families may allow this thyasirid clam to resist the toxic effect of extracellular symbiosis.

### Independent genome evolution drives the diversify of the deep-sea bivalve symbioses

Symbiosis is a major driven force of evolution, and this relationship shapes the phenotypic complexity of the symbiotic host [13, 85]. Among bivalves, chemosymbiosis with primary producers in the deep sea is one of the convergent adaptations to the oligotrophic environmental context [7]. However, symbiosis-related phenotypes, such as the spatial structure of bacteriocytes and the physiological adaptations between the host and the symbiont, are highly variable in bivalves [25]. The evolutionary fingerprints under these variations need further investigation.

Symbiont digestion through phagolysosome contributes significantly to the nutritional transfer from the symbiont to the host, and evidence has been obtained from both mussels and clams [33, 34]. However, as revealed by genomic comparisons, gene families related to symbiont digestion were divergently evolved among the three symbiotic bivalves. The expansion events in *C. bisecta* may enhance the phagocytic capabilities of this clam. For instance, cathepsins are representative hydrolases that participate in symbiont digestion, and the gene duplication of *ctsL* likely facilitated its specific expression in the symbiotic gill tissue. Regarding substrate transport from the host to the symbionts, genetic divergences were also observed between the extracellular symbiotic thyasirid and its endosymbiotic relatives. Thus, these divergences in currency exchange might account for the distinct spatial structure of bacteriocytes among symbiotic bivalves, whereas these evolutionary events converged to the facilitating of currency exchange.

The immune systems of the three symbiotic bivalves were remodeled at the genome level, but the evolutionary patterns were distinct. For instance, the bacterial LPS is one of the most potent initiators of inflammation and toxic to bivalves [80, 81]. According to our analysis, the symbiotic bivalves employed different pathways to adapt to the long-term co-existence with their bacterial symbionts. For example, expanded IAP families in the endosymbiotic bivalves have been linked to the homeostatic maintenance in bacteriocytes [22, 26]. Instead, we found that the expansion events in the LPS scavenging pathway might account for the thyasirid’s adaptation to the bacteria-hosting life. Likewise, although the *G. platifrons* and *C. bisecta* recruit symbiont horizontally and show a high degree of specificity with the symbiont, the bacterial recognition-related gene expansion events were divergent.

Our results suggest that distinct genome events, in particular gene duplications, underlie the independent evolution of symbiosis by deep-sea bivalves. Gene duplication results from stochastic accidents during inheritance and is considered a rich source of evolutionary substrates [86, 87]. The emerging evolutionary picture from our data is that gene duplications occurred persistently and stochastically, either before or after the establishment of symbiosis in the ancestors of symbiotic bivalves. During the many millions of years of co-evolution between the bivalves and their symbionts, selective pressure has resulted in extant species with optimal currency exchange and a high-tolerance immune strategy.

## Conclusions

Biologists have long been fascinated by the evolution of symbiotic relationships. Bivalves have shown varied symbiosis forms, and phenotypic divergence-driven comparison of bivalve genomes may provide insights into this question. By characterizing the hologenome of *C. bisecta*, genetic clues for the nutritional complementarity and immune interactions between the host and the Thioglobaceae symbiont were provided. Furthermore, by comparing this extracellular symbiotic thyasirid with endosymbiotic bivalves, we show that bivalves employ divergent evolution of genes and pathways, including phagocytosis, bacterial recognition, and immune response to LPS, to adapt to the long-term co-existence with their bacterial symbionts, highlighting the contribution of stochastic evolution to the independent gain of a symbiotic lifestyle in the lineage. Thus, our work provides a resource and valuable insights for understanding the evolution and dynamics of invertebrate symbiosis.

## Methods

### Sample collection

Individuals of *Conchocele bisecta* were collected during R/V *Kexue* “2018 Hydrothermal vent & Cold-seep combined expedition” at hydrothermal vents field in the East China Sea (27° 47.5′ N, 126° 53.8′ E), with the help of TV grab. Anatomy was processed on ice immediately after sampling, and tissues were stored at −80°C unless otherwise mentioned.

### FISH and TEM analysis

Both the gill tissue and gonad tissue of freshly collected clam were prepared for fluorescent in situ hybridization (FISH) analysis. In brief, the tissues were fixed in 4% paraformaldehyde at 4°C for 16 h. The fixed tissues were washed in cold PBS for three times and dehydrated in 100% methanol, and dehydrated tissues were stored at −20°C before use. The tissues were embedded with Paraplast plus (Sigma) first and cut into sections of 7-μm thickness with a microtome (Leica) for FISH analysis. Probe was labeled with Cy3 for FISH analysis and designed based on 16S rDNA sequence of the symbiont. The 16S-Probe was mapped to both the host genome and the assemblies of metagenomes of gill tissue to avoid unspecific hybridization. Sequential sections of gill or gonad tissue of *C. bisecta* were analyzed using both the 16S-Probe and the reverse complement one of it. The FISH experiments were performed according to the method described by Halary [88].

For transmission electron microscope (TEM) analysis, gill tissues were dissected and fixed in 2.5% glutaraldehyde and 2% paraformaldehyde at 4°C. Then the tissue was post-fixed with 1% osmium tetroxide and embedded in Ep812 resin. Sections of 70-nm thickness, which generated by a ultramicrotome (Reichert-Jung ULTRACUT E), were double-stained with lead citrate and uranyl acetate. The sections were examined using a TEM (JEM1200, JEOL) set to 100 kV.

### Amplicon sequencing of 16S rDNA V4 region

DNA templates that extracted from gill tissues of three individuals and gonad tissue of one individual were used for amplicon sequencing of 16S rDNA V4 region. The libraries were constructed and sequenced according to the instruction of BGISEQ-500 platform. Adapters and low-quality reads were removed, and operational taxonomic units (OTUs) were assigned to the SILVA ribosomal RNA database (r123) using the USEARCH pipeline with a 97% similarity cut-off. All OTUs mapped by more than 10 reads in any of the three samples were considered to be candidates of symbionts.

### Nucleic acid extraction and sequencing

Genomic DNA was extracted from the muscle tissue of *C. bisecta* using QIAamp DNA Mini Kit (Qiagen), and the DNA was examined with the Agilent 4200 Bioanalyzer (Agilent Technologies). Similarly, metagenomic DNA was exacted from tissues of gill and gonad by an identical progress, respectively.

For WGS sequencing, the DNA was fragmented to 500–800 bp in size with Covaris E220 and selected using AMPure XP beads to obtain fragments around 200 bp. Then the fragments were end-repaired and A-tailed with T4 DNA polymerase (NEB), T4 polynucleotide kinase (NEB), and *rTaq* DNA polymerase (Takara). After that, the DNA were amplified for eight PCR cycles and sequenced on the BGISEQ-500 platform with a layout of pair-end 100 bp.

For PacBio sequencing, 8 μg of extracted genomic DNA was sheared and concentrated with AMPure PB beads. The libraries were constructed using the Pacific Biosciences SMRTbell express template prep kit 2.0, and the constructed libraries were selected on a BluePippin system for molecules longer than 20 kb. Finally, the templates were primer annealed and bound to polymerases with the DNA/Polymerase Binding Kit, and sequencing was carried out on the Pacific Biosciences Sequel II platform for 15 h.

For Hi-C library construction, gonad tissue was dissociated, and cells were collected and crosslinked with 1% formaldehyde (Sigma) and 0.2M glycine (Sigma). After that, the fixed powder was resuspended in nuclei isolation buffer and then incubated in 0.5% SDS for 10 min at 62°C. Then the reaction was quenched with 10% Triton X-100 (Sigma) and the nuclei were collected by centrifugation. Then the DNA was digested with *MboI* (NEB), and the overhang was filled and biotinylated before ligated by T4 DNA ligase (NEB). Before library construction, the DNA was purified using the CTAB method. The purified DNA was sheared, and biotin-containing fragments were captured on streptavidin-coated beads using Dynabeads MyOne Streptavidin T1 (Invitrogen). Then the fragments were end-repaired and linked with adaptors before eight cycles of PCR reaction with KAPA HiFi HotStart ReadyMix (Kapa Biosystem). After that, the Hi-C library was sequenced with BGISEQ-500 platform with a layout of pair-end 100 bp.

For RNA sequencing, tissue-specific total RNA was extracted from tissues of adductor muscle, mantle, foot, and gill with TRIzol (Invitrogen), and the reverse transcription was performed with HiscriptII (Vazyme) to generate cDNA. The cDNA fragments were sequenced on the BGISEQ-500 platform with a layout of pair-end 100 bp.

For metagenome sequencing, selected DNA fragments were end-repaired, A-tailed, and amplified, and the libraries were sequenced on the BGISEQ-500 platform with a layout of pair-end 100 bp. In addition, DNA of gill tissues for Oxford Nanopore Technologies (ONT) long-read sequencing was extracted from gill tissues of a third individual with the Blood & Cell Culture DNA Midi Kit (Qiagen), and the library were prepared and sequenced with the Oxford Nanopore Ligation Sequencing Kits SQK-LSK109 according to the manufacturer’s instructions.

### Mitochondrial genome assembly and collinearity analysis

*Conchocele* cf. *bisecta* HPD1644 mitochondrial sequence [31], retrieved from the NCBI with sequence number “LC126312.1,” was used as the “Seed Input” in the configuration file of NOVOPlasty v4.2 [89], resulting in the mitochondrial genome of *C. bisecta* in this study. The genome was annotated using online tool of MITOS (http://mitos2.bioinf.uni-leipzig.de/index.py) [90]. For collinearity analysis, the assembled mitochondrial sequences were aligned to that of *Conchocele* cf. *bisecta* HPD1644 using Sibelia v2.1.1 [91] to generate a Circos configure file and plot in Circos v0.69 [92].

### Host genome assembly and chromosome anchoring

Before genome assembly, all of the generated short reads were quality filtered using SOAPnuke v1.5.2, with the parameter as “-l 20 -q 0.2 -Q 2 -d” [93]. K-mer frequency-based method was used for genome size estimation. In detail, the K-mers were counted using Jellyfish v2.2.6 with the parameter “-m 17”, and the output file was employed to estimate the genome size with “histo” of Jellyfish v2.2.6 [94].

The generated PacBio reads were converted to fasta format using bam2fastq. These raw reads were assembled by Shasta-OldLinux-0.4.0 with a PacBio-CLR configure file (minReadLength = 10000, maxAlignmentCount = 50, consensusCaller = Modal) [95]. This primary assembly was then polished twice using clean short reads generated by BGI sequencer by Pilon v1.22 [96]. BUSCO v5.3 estimation of the percentage of complete metazoan (odb10) dataset reached 87.8% [97]. Contigs from this assembly were then clustered using Hi-C data, after quality control process with HiC-Pro v3.2 [98] and assembled by 3D-DNA [99], the final heat map showed these contigs were mounted into 17 pseudo-chromosomes visualized in Juicebox v1.9 [100].

### Repeat annotation

Tandem Repeats Finder v 4.0.7 program was applied to detect tandem repeats in the genome [101]. Homolog-based and de novo prediction methods were integrated to identify transposable elements (TEs). For the de novo search, LTR_Finder v1.0.6 [102] and RepeatModeler v1.0.8 [103] were employed to find repetitive elements with specific consensus models. For the homology-based search, RepeatMasker v4.0.6 and RepeatProteinMask v4.0.6 against the Repbase v21.01 database were used at the nucleotide and protein levels respectively [104, 105]. Secondly, RepeatMasker was employed again to detect species-specific TEs against the database concatenated by the results of LTR_Finder and RepeatModeler together. All other species used in this work were annotated repetitive elements following the same pipeline for comparative analysis.

### Gene annotation

Ab initio, homology-based and gene expression evidence were combined to predict protein-coding genes in the genome of *C. bisecta*. Augustus v3.1 was first employed on repeat-masked genome for ab initio gene prediction [106]. For the homology-based annotation, gene sets from 10 molluscan species (*Archivesica marissinica*, *Biomphalaria glabrata*, *Crassostrea gigas*, *Gigantidas platifrons*, *Lottia gigantea*, *Lutraria rhynchaena*, *Modiolus philippinarum*, *Octopus bimaculoides*, *Pinctada fucata*, and *P. canaliculate*) were used. These homologous protein sequences were first aligned onto the genome of *C. bisecta* using Blast v2.2.26 with an e-value cut-off of 1 × 10^−5^ [107], and then we linked the alignment hits to candidate gene *loci* by GenBlastA [108]. Secondly, genomic sequences of candidate gene regions together with their 2-kb flanking sequences were extracted and used GeneWise v2.2.0 to determine gene models [109]. Moreover, Stringtie v 1.3.4 was employed to generated gene annotation files on RNA-Seq alignments generated by HISAT v2.1.0 of different tissues (adductor muscle, mantle, foot, and gill) [110, 111]. Then these files were merged together to predict candidate coding regions open reading frames (ORFs) using Transdecoder v5.5.0 and were aligned to genomes to obtain a gene annotation file with transcript evidence. Finally, these three evidences were integrated using EVM v1.1.1 to obtain a final version of protein-coding genes [112], and their function were annotated by searching against the following public databases: Swiss-Prot v201709, KEGG v87.0, InterPro v55.0, and TrEMBL v201709. The other 7 species used in gene family analysis were functionally annotated in the same way.

### Phylogenetic relationships and divergence times of mollusks and selected bivalves

Twenty-four well-assembled lophotrochozoan genomes were selected for phylogenetic analysis, include one annelid (*Helobdella robusta*) as outgroup, 21 bivalves (*Archivesica marissinica*, *Argopecten concentricus*, *Argopecten irradians*, *Conchocele bisecta*, *Crassostrea gigas*, *Crassostrea virginica*, *Cyclina sinensis*, *Gigantidas platifrons*, *Lutraria rhynchaena*, *Mactra quadrangularis*, *Mercenaria mercenaria*, *Mizuhopecten yessoensis*, *Modiolus philippinarum*, *Mytilus coruscus*, *Pecten maximus*, *Pinctada fucata*, *Pinctada imbricata*, *Ruditapes philippinarum, Saccostrea glomerata*, *Scapharca broughtonii, Sinonovacula constricta*), 5 gastropods (*Aplysia californica*, *Chrysomallon squamiferum, Lottia gigantea*, *Haliotis rufescens*, *Pomacea canaliculata*), and 2 cephalopods (*Octopus bimaculoides* and *Octopus vulgaris*) [22, 26, 52, 113–132]. SonicParanoid v1.3.0 was used to define gene family clusters among different species [133]. The amino acid sequences of one-to-one single-copy orthologous genes were used to reconstruct their phylogenetic topology. The protein sequences were aligned using MAFFT v7.407 under default settings [134], and then were concatenated for phylogenetic analysis using a maximum-likelihood method implemented in IQ-TREE v 2.0.6 with the “-m MFP” parameter was applied to each protein partition [135]. To estimate divergence times, the rooted maximum-likelihood tree, along with a concatenated fourfold degenerate site sequence extracted from single-copy CDS (coding sequence), was used as the input of MCMCtree software implemented in PAML v4.8 [136]. For calibration, nine nodes were constrained by either fossil records obtained from website of TimeTree.

### Host gene family analysis and domain analysis

For expansion and contraction analysis, in addition to genome of *C. bisecta*, we selected 7 representatives with good BUSCO performance out of the 15 collected bivalve genomes. The selected genomes include that of the only two published endosymbiotic bivalves (*A. marissinica*, *G. platifrons*) [22, 26], and 5 asymbiotic bivalves (*Crassostrea gigas*, *L. rhynchaena*, *M. yessoensis*, *Modiolus philippinarum*, *P. fucata*) which were separated in different bivalve clades and not known to host chemosynthetic bacteria. Before analysis, HMMSCAN (HMMER v3.1) was applied to identify Pfam domains in protein-coding gene sequences among the selected bivalve. The Pfam domains of the respective species were counted to construct a data frame, while multiple copies of a same domain in the same gene were counted as one.

Gene family analyses in the symbiotic bivalves were conducted using one-tailed Fisher’s exact tests for either expansion or contraction. In detail, for gene expansion/contraction at the protein domain level, we first calculated the counts of each Pfam domain in each genome of the 8 analyzed species, and the Pfam domain counts in each of the symbiotic bivalves (*A. marissinica*, *C. bisecta*, *G. platifrons*) was compared against the background average domain counts of the five asymbiotic bivalve genomes (*Crassostrea gigas*, *L. rhynchaena*, *M. yessoensis*, *Modiolus philippinarum*, *P. fucata*), which method was employed by Sun et al. for comparative genomic analysis [26]. Furthermore, we conducted the same analysis with the gene counts of each KEGG ortholog on each of the three symbiotic bivalves. After that, Pfam domain or KEGG ortholog with a *P* value less than 0.05 is considered statistically expanded or contracted in the three symbiotic bivalves. Finally, the evolutionary patterns of *A. marissinica*, *C. bisecta*, and *G. platifrons* were compared according to the expansion/contraction results by Fisher’s exact tests.

For phylogenetic analysis of each gene family, we employed Muscle v3.8.31 for multiple sequence alignment [137], and the phylogenetic trees were constructed with FastTreeMP v2.1.10 [138]. Specially, for reported expansion events of subfamilies of hemoglobin, which were not included in the KEGG database, we performed additional alignment with the sequences mentioned in Ip et al. [22] using Diamond, and phylogenetic analysis was conducted as the same.

### Time of gene duplication

To evaluate the temporal dynamics of expanded gene families during the evolution of *C. bisecta*, the nucleotide substitution rates of bivalves were calculated by the branch distance divided by the estimated divergence time using MCMCtree. With default settings of MAFFT and “-automated1” option of trimAl v1.4 [139], all paralogs of the target gene family were aligned to determine the time required for gene duplication. The Nei-Gojobori pairwise codeml method was used to determine the d*N* values for all aligned pairs. Divergence times of gene pairs were estimated using the equation *T* = *K*/2*r* [140], where *T* is the insertion time, and *r* is the nucleotide substitution rate. The relationships between different gene pairs are determined following the DupGen_Finder (https://github.com/qiao-xin/DupGen_finder) pipeline, using *Nematostella vectensis* as a reference genome.

### Transcriptome analysis

Adaptor sequence removal and read quality filtering from the raw transcriptome datasets were performed using SOAPnuke, with the parameter as described above. HISAT2 were used to build index and align clean RNA reads to the reference genome. RNACocktail v0.2.1 were used to build genome-guided transcriptome assemblies [141]. Tissue-specific expression levels were quantified using Salmon v0.8.2 [142].

### Metagenomic assembly and binning

The raw reads generated from either metagenomic libraries of the two individual gill tissues were filtered using SOAPnuke. The filtered reads which were confidently aligned to the host’s genome were removed, and remained reads were assembled with MEGAHIT v1.1 (--min-count 2/3 --k-min 33 --k-max 53 --k-step 10 --no-mercy) [143]. Metagenome-assembled genomes (MAGs) were binned with Metawrap v1.1.5 [144], and the bins were combined and filtered with the “bin_refinement” module from Metawrap. To further completing the MAGs, all metagenomic short reads were mapped to each MAGs, and we employed the hybrid assembler Unicycler v0.4.8 to de novo assemble the genomes with both the mapped short reads and all of the ONT reads [145]. Finally, we calibrated the MAGs again by using the GATK pipeline to remove potential errors, and the MAGs’ taxonomic assignment was completed with GTDB-tk v1.0.2 [146, 147]. CheckM v1.0.12 was used to estimate the completeness and contamination of the genomes [148].

To annotate the symbiont genome, we employed Prokka v1.14.6 to predict the CDS region [149], and tRNAscan-SE v1.3.1 to identify the tRNA gene [150], and RNAmmer v1.2 were used to identify the rRNA gene for annotation [151]. In parallel, Diamond was employed to align the protein sequences to the KEGG. Transporters were predicted by online tools of TransAPP and ABCdb [41, 42]. Metabolic potential of the bacterial symbiont in amino acids and cofactors were checked thoroughly, and missed genes were confirmed by aligning the ONT long reads to the chromosomal level genome of endosymbiont of *Bathymodiolus septemdierum* (NCBI Accession Number: NZ_AP013042.1), while the results were visualized in IGV v2.10 [152].

### Metaproteomics

The gill tissue was grinded and sonicated using a high-intensity ultrasonic processor (Scientz). After centrifugation at 4°C, the supernatant was collected and the protein concentration was determined with BCA kit according to the manufacturer’s instructions. Next, the protein solution was reduced with 5 mM dithiothreitol for 30 min at 56°C and alkylated with 11 mM iodoacetamide for 45 min at room temperature in darkness, and trypsin was employed to digest the protein. Tryptic peptides were dissolved and separated on a nanoElute UHPLC system (Bruker Daltonics). The peptides were subjected to mass spectrometry with the timsTOF Pro (Bruker Daltonics) in a parallel accumulation serial fragmentation (PASEF) mode. With 1.65 kV, precursors and fragments were analyzed at the TOF detector with settings including Tandem Mass Spectrometry (MS/MS) scan range from 100 to 1700 m/z, precursors with charge states 0 to 5 for fragmentation, 10 PASEF-MS/MS scans per cycle, and 30s for dynamic exclusion. The data were processed with MaxQuant search engine v.1.6.15.0. Tandem mass spectra were searched against the protein catalog of the hologenome (26928 entries) concatenated with reverse decoy database. The mass tolerance for precursor ions was 20 ppm and that for fragment ions was 0.02 Da, and FDR (false discovery rate) was adjusted to 0.01. The iBAQ values of the gill tissue were normalized as iBAQ values per million.

## Supplementary Information

**Additional file 1: Supplementary Note 1.** Taxonomic identification of the bivalve samples. **Supplementary Note 2.** Metabolic potential of SCbi. **Supplementary Note 3.** The genome assembly of *Conchocele bisecta*. **Supplementary Note 4.** Transporters for B type vitamins in *C. bisecta*. **Supplementary Note 5.** Filter-feeding related gene families in *C. bisecta*. **Figure S1.** Shells and mitochondrial genome of *C. bisecta*. **Figure S2.** Fluorescence in situ hybridization (FISH) of 16S rRNA of the dominate symbionts in the gill filaments. **Figure S3.** Biosynthesis pathways of amino acids in *C. bisecta* and its symbionts. **Figure S4.** Biosynthesis pathways of cofactors in *C. bisecta* and its symbionts. **Figure S5.** Alignment results by mapping the ONT reads of SCbi to a published SUP05 genome. **Figure S6.** Expression of genes involved in biosynthesis of threonine in both the symbiont and its *C. bisecta* host. **Figure S7.** Expression of genes involved in biosynthesis and transport of folate in both the symbiont and its *C. bisecta* host. **Figure S8.** The assembly of the genome of *C. bisecta*. **Figure S9.** Chromosome-scale macro-synteny comparison between *Nematostella vectensis* and six bivalves. **Figure S10.** Relationship between genome size and transposable elements (TE) in the lophotrochozoan genomes. **Figure S11.** Expanded or contracted Pfam domains in *C. bisecta*. **Figure S12.** Expression levels of genes related in transport of rare metabolites in *C. bisecta*. **Figure S13.** Carbonic anhydrases in bivalves. **Figure S14.** Hemoglobin and hemoglobin-like proteins in bivalves. **Figure S15.** Expression levels of expanded genes that implicated in phagocytosis in *C. bisecta*. **Figure S16.** Expression levels of expanded genes that implicated in recognition and homeostasis in *C. bisecta*. **Table S2.** Genomic statistics of the SCbi (Symbionts of *C. biescta*). **Table S5.** Distribution genes involved in amino acids biosynthesis in SUP05 bacteria. **Table S6.** Statistics of sequenced genomic data. **Table S7.** Characteristics of the *C. bisecta* genome assembly. **Table S8.** The mapping rate of short reads to *C. bisecta* genome. **Table S9.** Chromosome statistics of *C. bisecta* genome. **Table S10.** TE contents (%) of lophotrochozoan genomes.

**Additional file 2: Table S1.** Constitution of prokaryotes in the gill of *C. bisecta* based on 16S rDNA amplicon analysis. **Table S3.** Putative transproter genes in SCbi. **Table S4.** Genes in biosynthesis pathways of amino acids and cofactors. **Table S11.** The expansion and contraction events among the three symbiotic bivalves (*Conchocele bisecta*, *Archivesica marissinica*, *Gigantidas platifrons*). **Table S12.** Counts of pfam domains related to the glycosyl hydrolase families in bivalve genomes. **Table S13.** Number of Pfam protein families related to the PRRs that involved in bacterial recognition.

## Data Availability

The assemblies and protein sequences of published genome used in the present study were from the NCBI repository: PRJNA629898 for *Archivesica marissinica* [22], PRJNA828432 for *Crassostrea gigas* [115], PRJNA328542 for *Gigantidas platifrons* [26], PRJNA548223 for *Lutraria rhynchaena* [121], PRJNA259405 for *Mizuhopecten yessoensis* [52], PRJNA328544 for *Modiolus philippinarum* [26], PRJNA283019 for *Pinctada fucata* [120]. All sequencing data for amplicons, genome, transcriptome, and metagenome, as well as the assemblies of both *Conchocele bisecta* and its bacterial symbiont, are available in the NCBI (National Centre for Biotechnology Information) repository under project PRJNA913320 [153]. In addition, all above datasets can also be found at CNGB Sequence Archive (CNSA) of the China National GeneBank DataBase (CNGBdb) under project CNP0003505 [153]. The metaproteomic data of *C. bisecta* was deposited to the Proteomics Identification (PRIDE) database under the accession number PXD036936 [154]. Genome assemblies and annotations of the *C. bisecta* hologenome are available in Figshare [155]. All computer commands are provided at Github [156].

## Abbreviations

Ad: Adductor
ALG: Ancestral linkage group
ApoH: Apolipoprotein H
Arcmar: *Archivesica marissinica*
b: Bacterial symbionts
BUSCO: Benchmarking universal single-copy orthologs
CCA: Cytoplasmic carbonic anhydrase
CCKAR: Cholecystokinin receptor
CDS: Coding sequence
CHRNA: Acetylcholine receptor
CI: Conservation index
CNGBdb: China National GeneBank DataBase
CNSA: CNGB Sequence Archive
Conbis: *Conchocele bisecta*
Cragig: *Crassostrea gigas*
CSTL: Cathepsin L
FDR: False discovery rate
FISH: Fluorescence in situ hybridization
Fo: Foot
GCNT1: Acetylglucosaminyltransferase
Gi: Gill
Gigpla: *Gigantidas platifrons*
GTDB: Genome Taxonomy Database
Hb: Hemoglobin
IAP: Inhibitor of apoptosis protein
iBAQ: Intensity-based absolute quantification
KO: KEGG ortholog
LITAF: Lipopolysaccharide induced TNF factor
LPS: Lipopolysaccharide
LRP: Low-density lipoprotein receptor-related protein
Lutrhy: *Lutraria rhynchaena*
Ma: Mantle
MAG: Metagenome-assembled genome
MCOLN: Mucolipin
Mizyes: *Mizuhopecten yessoensis*
Modphi: *Modiolus philippinarum*
MPR: Mannose-6-phosphate receptor
MS/MS: Tandem mass spectrometry
mv: Microvilli
MY: Million years
MYA: Million years ago
n: Nucleus
NCBI: National Centre for Biotechnology Information database
ONT: Oxford Nanopore Technologies
ORF: Open reading frame
OTU: Operational taxonomic unit
PASEF: Parallel accumulation serial fragmentation
Pinfuc: *Pinctada fucata*
pl: Phagolysosome-like organelles
PRIDE: Proteomics Identification database
PRR: Pattern recognition receptor
Rab35: Ras-related protein Rab-35
Rac1: Rac family small GTPase 1
RNF213: E3 ubiquitin-protein ligase ring finger protein 213
ROS: Reactive oxygen species
SCbi: The bacterial symbiont of *Conchocele bisecta*
Stx7: Syntaxin7
TE: Transposable element
TEM: Transmission electron micrography
TNF: Tumor necrosis factor
TPM: Transcript per million
v: Vesicle
VB: B vitamins
VPS34: Phosphatidylinositol 3-kinase
