# Supplementary material for: Hologenome analysis reveals independent evolution to chemosymbiosis by deep-sea bivalves
Source: BMC Biol. 2023 Mar 8;21:51. doi: 10.1186/s12915-023-01551-z (PMC9993606; doi:10.1186/s12915-023-01551-z)
Supplement: Supplementary file 1 — Additional file 1: Supplementary Note 1. Taxonomic identification of the bivalve samples. Supplementary Note 2. Metabolic potential of SCbi. Supplementary Note 3. The genome assembly of Conchocele bisecta. Supplementary Note 4. Transporters for B type vitamins in C. bisecta. Supplementary Note 5. Filter-feeding related gene families in C. bisecta. Figure S1. Shells and mitochondrial genome of C. bisecta. Figure S2. Fluorescence in situ hybridization (FISH) of 16S rRNA of the dominate symbionts in the gill filaments. Figure S3. Biosynthesis pathways of amino acids in C. bisecta and its symbionts. Figure S4. Biosynthesis pathways of cofactors in C. bisecta and its symbionts. Figure S5. Alignment results by mapping the ONT reads of SCbi to a published SUP05 genome. Figure S6. Expression of genes involved in biosynthesis of threonine in both the symbiont and its C. bisecta host. Figure S7. Expression of genes involved in biosynthesis and transport of folate in both the symbiont and its C. bisecta host. Figure S8. The assembly of the genome of C. bisecta. Figure S9. Chromosome-scale macro-synteny comparison between Nematostella vectensis and six bivalves. Figure S10. Relationship between genome size and transposable elements (TE) in the lophotrochozoan genomes. Figure S11. Expanded or contracted Pfam domains in C. bisecta. Figure S12. Expression levels of genes related in transport of rare metabolites in C. bisecta. Figure S13. Carbonic anhydrases in bivalves. Figure S14. Hemoglobin and hemoglobin-like proteins in bivalves. Figure S15. Expression levels of expanded genes that implicated in phagocytosis in C. bisecta. Figure S16. Expression levels of expanded genes that implicated in recognition and homeostasis in C. bisecta. Table S2. Genomic statistics of the SCbi (Symbionts of C. biescta). Table S5. Distribution genes involved in amino acids biosynthesis in SUP05 bacteria. Table S6. Statistics of sequenced genomic data. Table S7. Characteristics of the C. bisecta genom [file 12915_2023_1551_MOESM1_ESM.docx]

*Supplementary information for:*

**Hologenome analysis reveals independent evolution to chemosymbiosis by deep-sea bivalves**

Content

[Supplementary Note 1 Taxonomic identification of the bivalve samples 2](#_Toc127954278)

[Supplementary Note 2 Metabolic potential of SCbi 2](#_Toc127954279)

[Supplementary Note 3 The genome assembly of *C. bisecta* 4](#_Toc127954280)

[Supplementary Note 4 Transporters for B type vitamins in *C. bisecta* 4](#_Toc127954281)

[Supplementary Note 5 Filter-feeding related gene families in *C. bisecta* 5](#_Toc127954282)

[Supplementary Figure 1 6](#_Toc127954283)

[Supplementary Figure 2 7](#_Toc127954284)

[Supplementary Figure 3 8](#_Toc127954285)

[Supplementary Figure 4 9](#_Toc127954286)

[Supplementary Figure 5 10](#_Toc127954287)

[Supplementary Figure 6 11](#_Toc127954288)

[Supplementary Figure 7 12](#_Toc127954289)

[Supplementary Figure 8 13](#_Toc127954290)

[Supplementary Figure 9 14](#_Toc127954291)

[Supplementary Figure 10 16](#_Toc127954292)

[Supplementary Figure 11 18](#_Toc127954293)

[Supplementary Figure 12 19](#_Toc127954294)

[Supplementary Figure 13 20](#_Toc127954295)

[Supplementary Figure 14 22](#_Toc127954296)

[Supplementary Figure 15 23](#_Toc127954297)

[Supplementary Figure 16 24](#_Toc127954298)

[Supplementary Table 2 25](#_Toc127954299)

[Supplementary Table 5 26](#_Toc127954300)

[Supplementary Table 6 27](#_Toc127954301)

[Supplementary Table 7 28](#_Toc127954302)

[Supplementary Table 8 29](#_Toc127954303)

[Supplementary Table 9 30](#_Toc127954304)

[Supplementary Table 10 31](#_Toc127954305)

# Supplementary Note 1 Taxonomic identification of the bivalve samples

The bivalve samples in this study were assigned to the species of *C. bisecta* based on both morphological characteristics and mitochondrial DNA (mtDNA) similarities. Firstly, several features (Fig. S1a, b) observed here were identical to that of species belongs to *Conchocele* (30), such as the inequilateral shells with prosogyrous beaks closed to the anterior; steeply sloped anterior margin; and large, deep, angulated posterior sulcus. Furthermore, a 17014 bp mitochondria genome (Fig. S1c) was assembled, and the COI gene shared an 99.93% identity to that of *C.* cf. *bisecta* HPD1644 (Fig. S1d) (31).

# Supplementary Note 2 Metabolic potential of SCbi

The SCbi encodes complete pathways for biosynthesis of 18 amino acids but threonine and histidine (Table S4, Fig. S3). Although we failed to locate *argH* (argininosuccinate lyase), *lysA* (diaminopimelate decarboxylase) and *leuB* (3-isopropylmalate dehydrogenase) in the SCbi genome, we found that these genes were mostly harbored by published SUP05 genomes (Table S5), and large amounts of our ONT reads were mapped to the loci of *argH*, *lysA* and *leuB* in the chromosomal level SUP05 genome with high confidences (Fig. S5). Thus, the SCbi should be able to synthesize the arginine, leucine and lysine. However, the *hisB* gene in the symbiont genome was annotated to the KEGG ortholog K01693, which was thought to be deficient in histidinol phosphate phosphatase activity (45), rendering the route to histidine incomplete. In addition, we failed to locate *thrB* (homoserine kinase) in all analyzed genomes of both symbiotic and free-living SUP05 bacteria. According to the proteomic data, other genes involved in the biosynthesis of threonine were highly expressed (Fig. S6), and similar pattern has been reported in endosymbionts in *A. marissinica*, emphasizing uncharacterized ways to synthesize threonine in these bacteria (22).

In addition, gene repositories for biosynthesis of ten cofactors (thiamine, riboflavin, pyridoxine, pantothenate, coenzyme A, NAD+, biotin, folate, siroheme and heme) were found in the SCbi genome, indicating that the SCbi may potentially provide cofactors to the host (Table S3, Fig. S4). Although *hemC* (hydroxymethylbilane synthase) was absent in the SCbi genome, but like *argH*, *lysA* and *leuB*, this gene was found in other published SUP05 genomes (Table S5), and the presents in SCbi was supported by ONT reads mapping (Fig. S5). Furthermore, *thiIF* (uracil 4-sulfurtransferase, ThiS adenylyltransferase), *epd* (D-erythrose 4-phosphate dehydrogenase), *panG* (2-dehydropantoate 2-reductase), and *ALP* (alkaline phosphatase) were absent in the symbiont genome. In SCbi, the depleted reactions may be compensated by alternative enzymes or intermediates from the host. For instance, the gene coding ALP was lost in all analyzed SUP05 genomes including SCbi. But the dihydroneopterin aldolase, whose substrate was dihydroneopterin, was highly translated, indicating that the existence of this ALP product (Fig. S7). Some clues indicated that the demand in dihydroneopterin of SCbi might be satisfied by the host. Several genes coding ALP were expressed in gill tissues of the host, and the expression level of host ALP was higher than those of genes coding the upstream GTP cyclohydrolase IA and downstream folylpolyglutamate synthase. Moreover, equilibrative nucleoside transporter, which shuttles dihydroneopterin in and out of cells (46), was expressed in the gill tissue (Fig. S7). However, we would not rule out uncharacterized bypass of this reaction in these SUP05 bacteria, since we have not found any related transporters expressed in the symbionts.

# Supplementary Note 3 The genome assembly of *C. bisecta*

Several approaches were combined to obtain a high-quality reference genome assembly of *C. bisecta* (Table S6). Approximately 320 G (168×) continuous long reads (CLR) of 2 cells generated by PacBio Sequel II platform were assembled into contigs. After two times of polishing, the ~1.9 Gb draft assembly, which was approximately equal to the estimated genome size of 2.1 Gb by k-mer analysis (Fig. S8), contained 17,791 contigs with a contig N50 length 488,582 bp (Table S7). The integrity of the assembly is demonstrated by 97.88% of sequencing reads mapping (Table S8). About 16.30% of in total ~210× Hi-C data were kept as valid reads after filtering, and based on these data, 97.0% of the scaffolds were successfully anchored to 17 lineage groups (Fig. 2a, Fig. S8, Table S9). BUSCO (Benchmarking Universal Single-Copy Orthologs) assessment of *C. bisecta* showed that it achieved 92.5% (87.8% complete and 4.7% fragmented) of the 978 universal single-copy orthologous metazoan genes (Table S7). With the integration of multiple evidence, whole-genome annotation articulates 25,483 protein-coding genes with 88.1% complete and 7.3% fragmented BUSCOs were attained using the metazoan database (Table S7).

# Supplementary Note 4 Transporters for B type vitamins in *C. bisecta*

The thyasirid host seems to only be able to synthesize the vitamin B6 among all B vitamins (Table S4, Fig S4). According to this, almost all necessary transporters known to transport B vitamins were found in genome of *C. bisecta*. Among these transporters, CUBN and SLC5A6 are responsible for absorption of VB12 and VB7, VB5, respectively. In line with the actively transcribed transporters of amino acid, we have found that genes belonged to these two families were highly expressed in the gill tissues of *C. bisecta* (Fig. S12). Thus, similar to what has been reported in previous studies, although some amino acids or co-factors cannot be synthesized in the thyasirid host, the highly expressed transporters have emphasized the nutritional function of the gill tissues.

# Supplementary Note 5 Filter-feeding related gene families in *C. bisecta*

It has been reported that thyasirids employed various feeding strategies, and many species are mixotrophs (28, 63). However, several evidences supported by the genome indicated that *C. bisecta* may largely relied on symbiosis for nutrition. The glycosyl hydrolase family (GHF) were found to be contracted in *A. marissinica* and *Paraescarpia echinospica*, which mostly if not totally relies on their symbionts (22, 64). Furthermore, the hydrolases are highly expressed in digestive tract of shallow water bivalves that filter-feed phytoplankton (65). Among all eight genomes compared, GHF domains were the least in genomes of *C. bisecta* (135 in total), while the average counts of the five asymbiotic bivalves is 190.2 (Table S12). Similar to the vesicomyid clam and the tubeworm, GHF5, GHF9, GHF10 and Glycosyl hydrolase catalytic core were relatively less in *C. bisecta* than the averages of the asymbiotic bivalves, or that of the shallow water clam, *L. rhynchaena* (Table S12). In addition, we failed to locate gene coding for either pancreatic elastase II (*cela2*) or chymotrypsin (*ctrb*), which two genes were coded in all the filter-fed bivalves, and *cela2* was also absent in genome of *A. marissinica*. Taken together, *C. bisecta* has lost gene families related in filter-feeding, and the symbiosis might be the major source of nutrient.

# Supplementary Figure 1

**
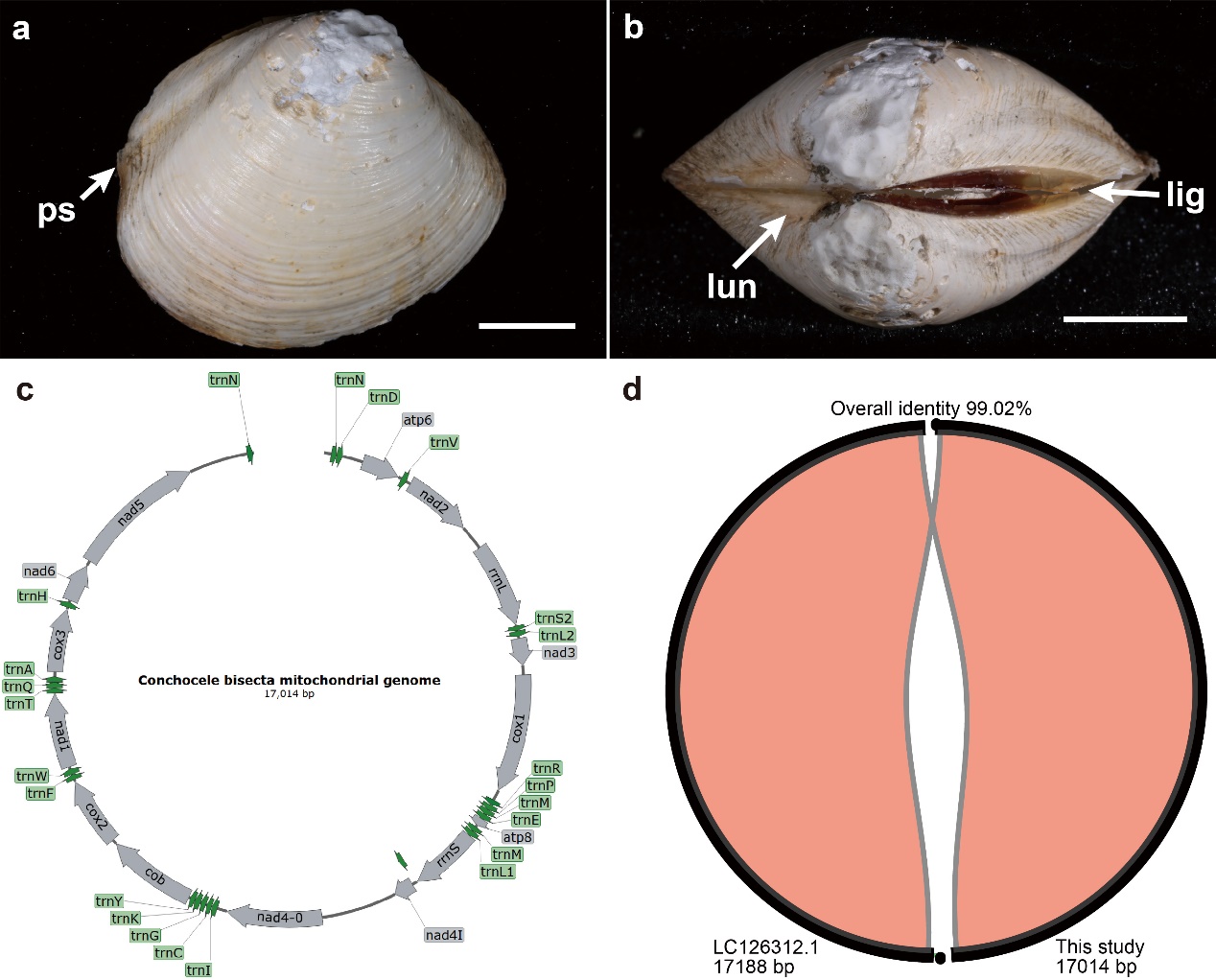
**

**Supplementary Figure 1** Shells and mitochondrial genome of *Conchocele bisecta*. a External view of right valve (scale bar: 10 mm), and the large, deep, angulated posterior sulcus(ps). b Dorsal view of the *C. bisecta* (lun: lunule; lig: ligament; scale bar: 10 mm). c Schematic representation of the *C. bisecta* mitochondrial genome. d Overall identity between the mitochondrial genomes in this study and published *C.* cf. *bisecta* HPD1644 (LC126312.1).

# Supplementary Figure 2


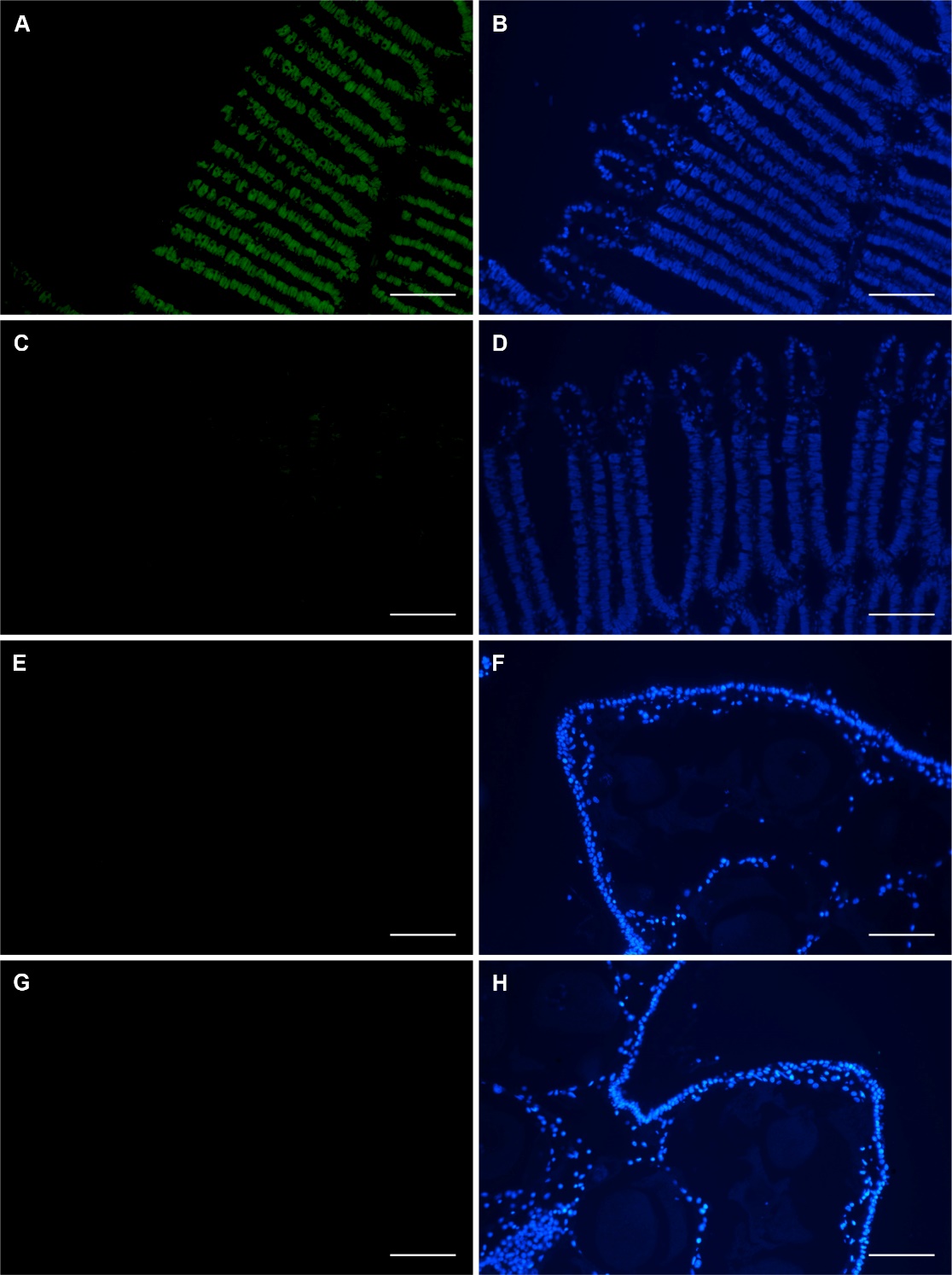


**Supplementary Figure 2** Fluorescence *in situ* hybridization (FISH) of 16S rRNA of the dominate symbionts in the gill filaments. The experiments were conducted with both gill tissue (A, B, C, D; scale bar: 100 μm) and gonand tissue (E, F, G, H; scale bar: 100 μm), and both the 16S rRNA probe (A, E) and its reverse complement sequence (negative control; C, G) were tagged with Cy3 dye. DNAs were stained with DAPI (B, D, F, H).

# Supplementary Figure 3


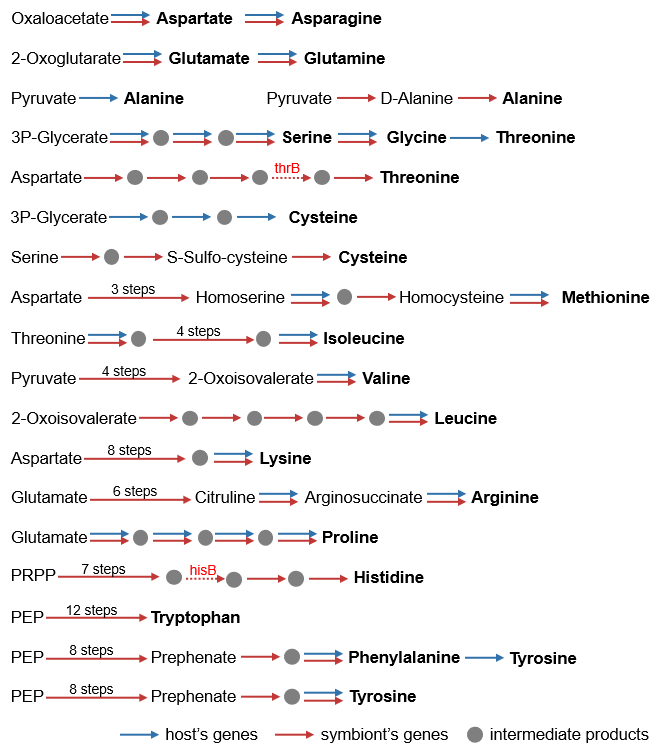


**Supplementary Figure 3** Biosynthesis pathways of amino acids in *Conchocele bisecta* (blue arrow) and its symbionts (red arrow). Missing genes in the symbionts were marked in red.

# Supplementary Figure 4


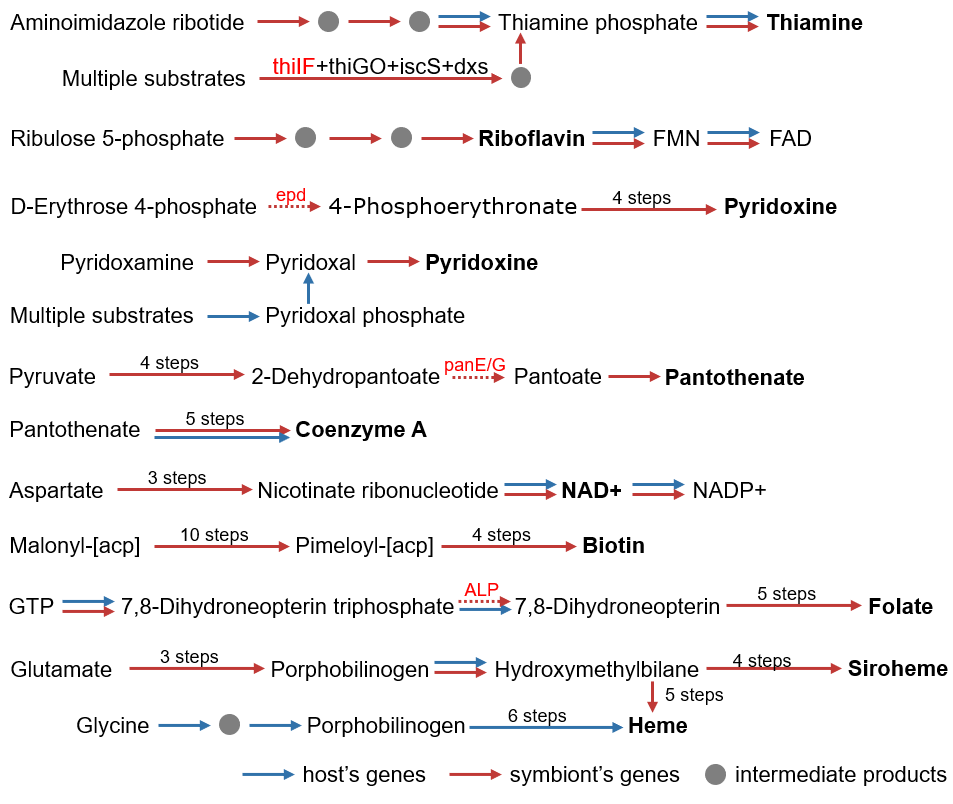


**Supplementary Figure 4** Biosynthesis pathways of cofactors in *Conchocele bisecta* (blue arrow) and its symbionts (red arrow). Missing genes in the symbionts were marked in red.

# Supplementary Figure 5


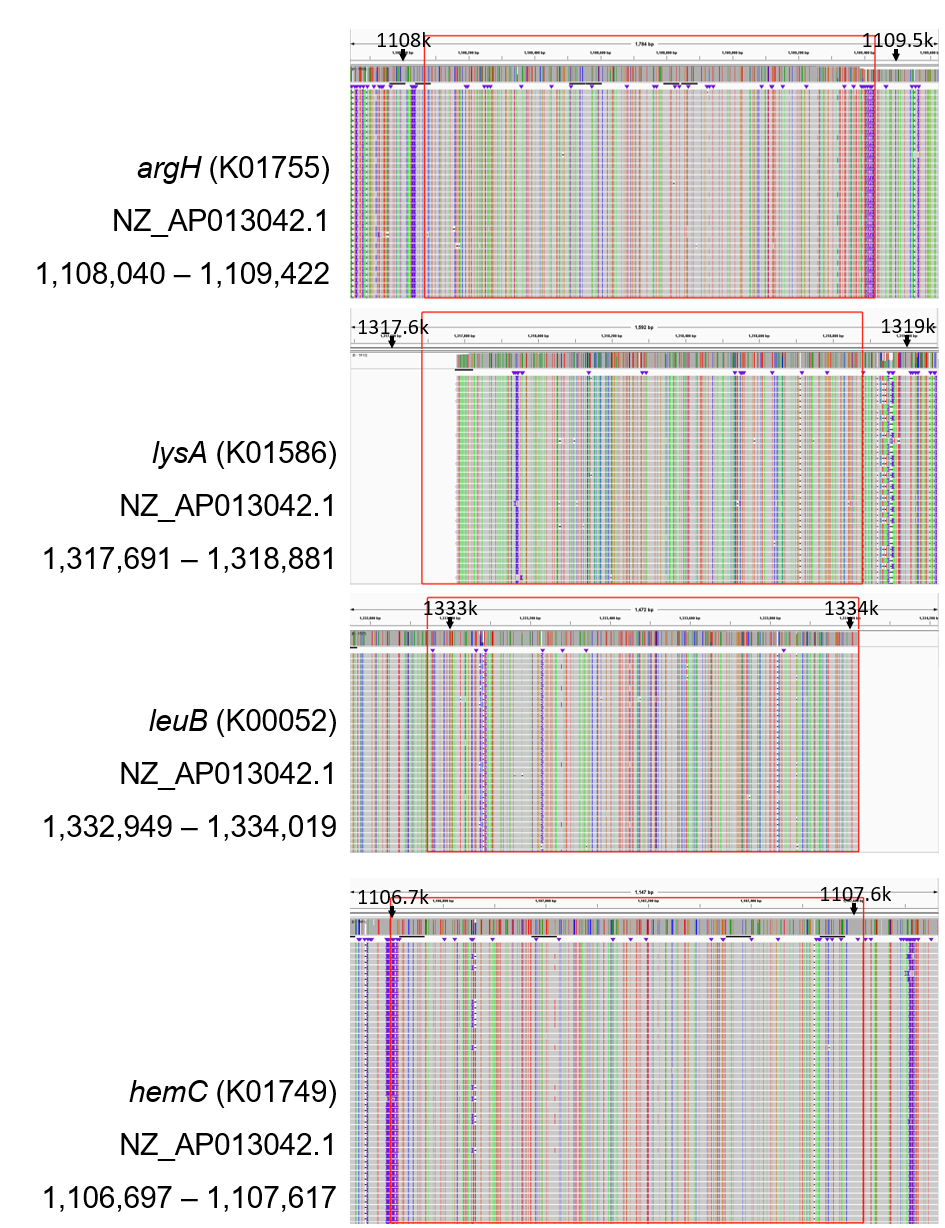


**Supplementary Figure 5** Alignment results by mapping the Oxford Nanopore Technologies (ONT) long reads of the symbionts of *Conchocele bisecta* to the coding region of *ArgH*, *LysA*, *LeuB* and *hemC* in a published chromosomal level SUP05 bacterial genome (NZ_AP013042.1).

# Supplementary Figure 6


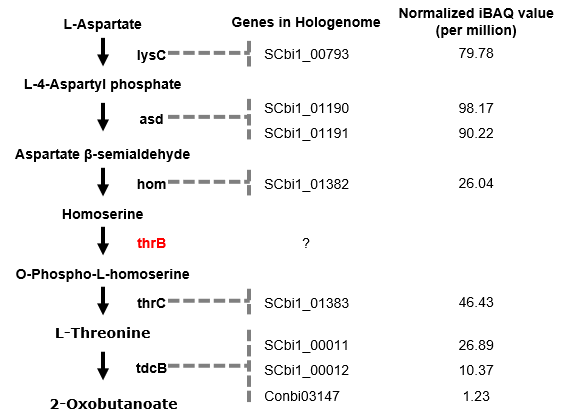


**Supplementary Figure 6** Expression of genes involved in biosynthesis of threonine in both the symbiont and its *Conchocele bisecta* host. The expression level were based on proteomic data, and all genes in the pathway of the symbionts (SCbi) and the host (Conbi) were actively expressed. Genes coding *thrB* (in red) are missed in the hologenome.

# Supplementary Figure 7


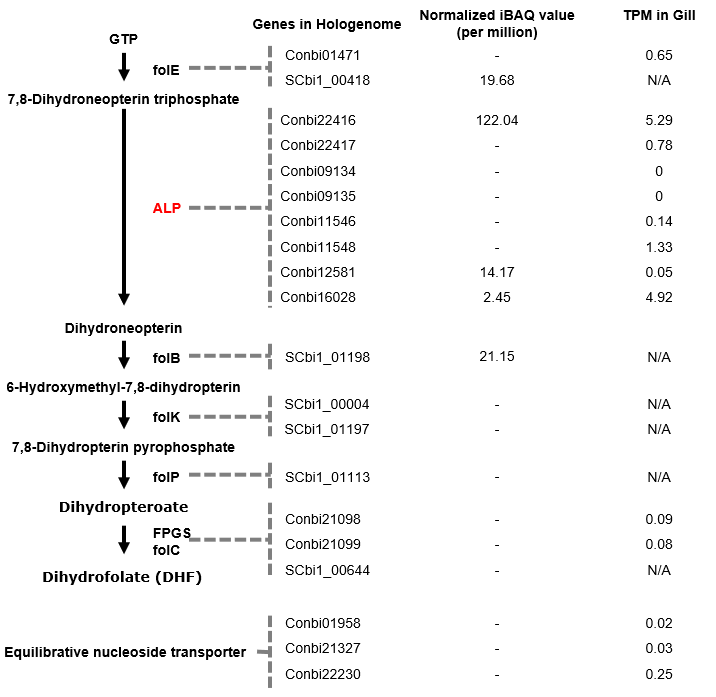


**Supplementary Figure 7** Expression of genes involved in biosynthesis and transport of folate in both the symbiont (SCbi) and its *Conchocele bisecta* host (Conbi). The expression level of the genes in symbiont were based on proteomic data, while the expression level of genes for the host were based on both proteomic and transcriptomic data. Genes coding alkaline phosphatase (ALP; in red) are missed in the genome of the symbionts, but the enzyme were actively expressed in host.

# Supplementary Figure 8


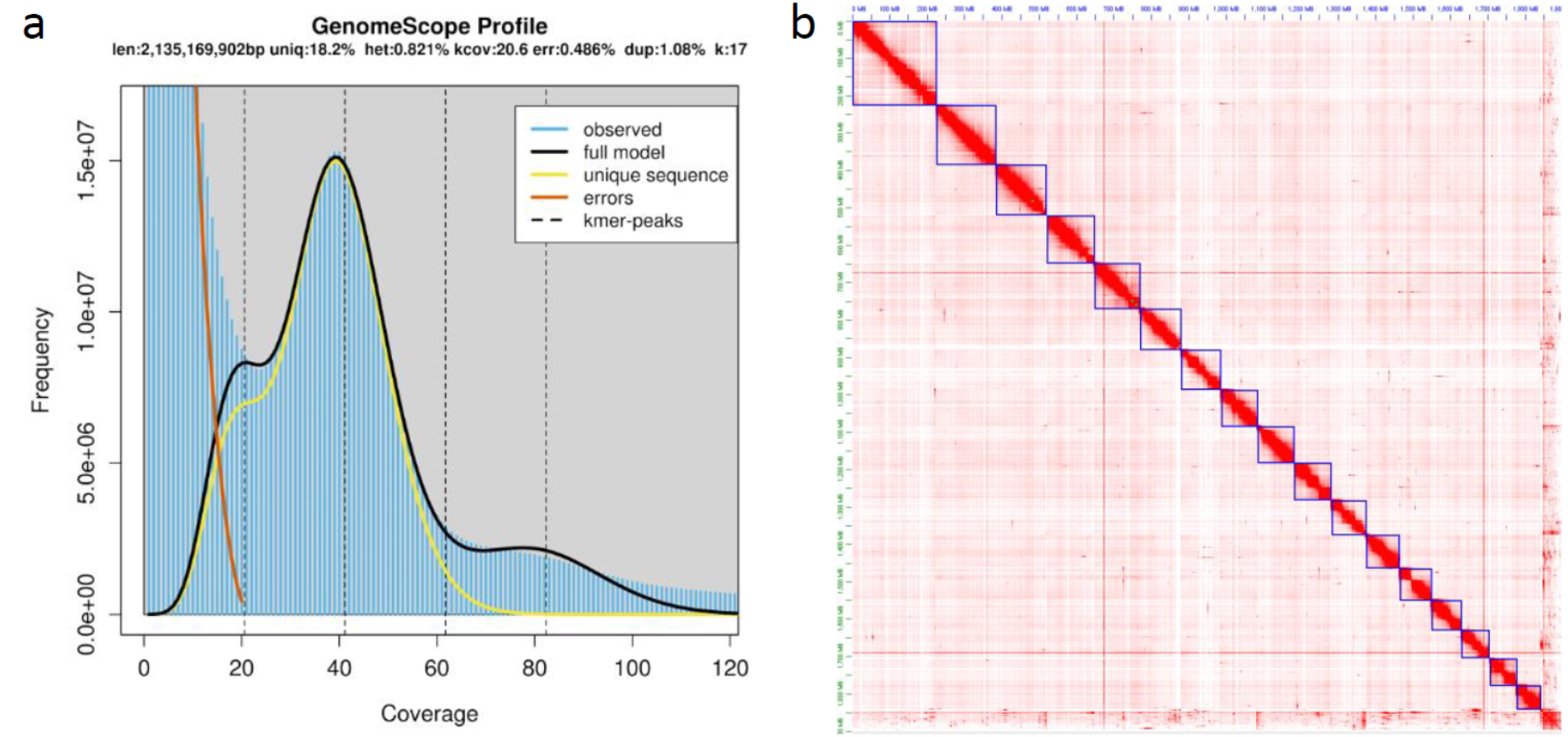


**Supplementary Figure 8** The assembly of the genome of *Conchocele bisecta*. a Estimation of genome size. The estimated genome size is ~2.1 Gb (k-mer: 17). b Genome-wide all-by-all Hi-C matrix. 97.0% of the scaffolds are anchored to 17 lineage groups.

# Supplementary Figure 9

**
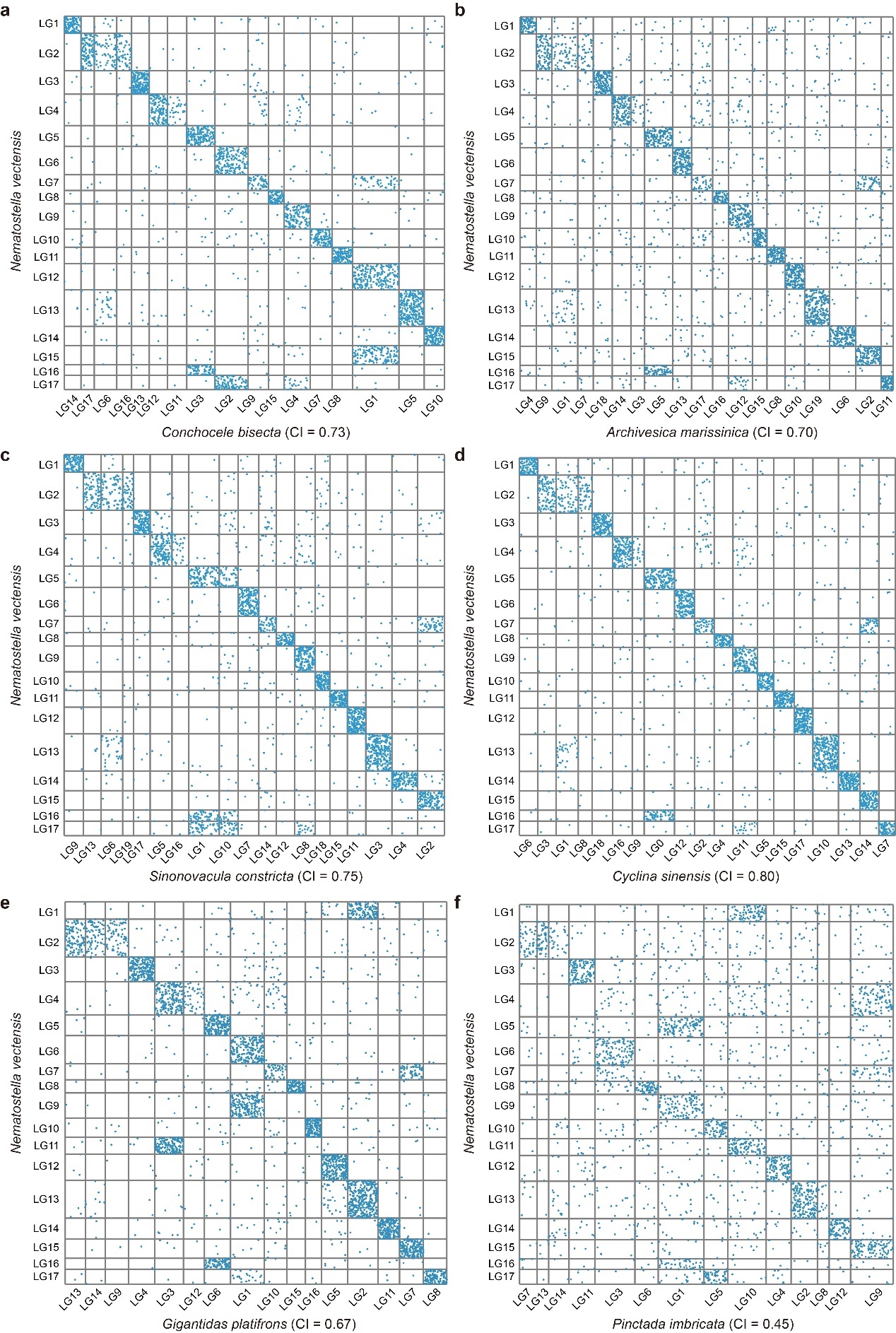
**

**Supplementary Figure 9** Chromosome-scale macro-synteny comparison between *Nematostella vectensis* and each bivalve of the six species: *Conchocele bisecta* (a), *Archivesica marissinica* (b), *Sinonovacula constricta* (c), *Cyclina sinensis* (d), *Gigantidas platifrons* (e), *Pinctada imbricata* (f). The conservation index (CI) of four bivalves with ancestral linkage groups (ALG) that represented by genes of *Nematostella vectensis* are shown in the bottom of each plot.

# Supplementary Figure 10


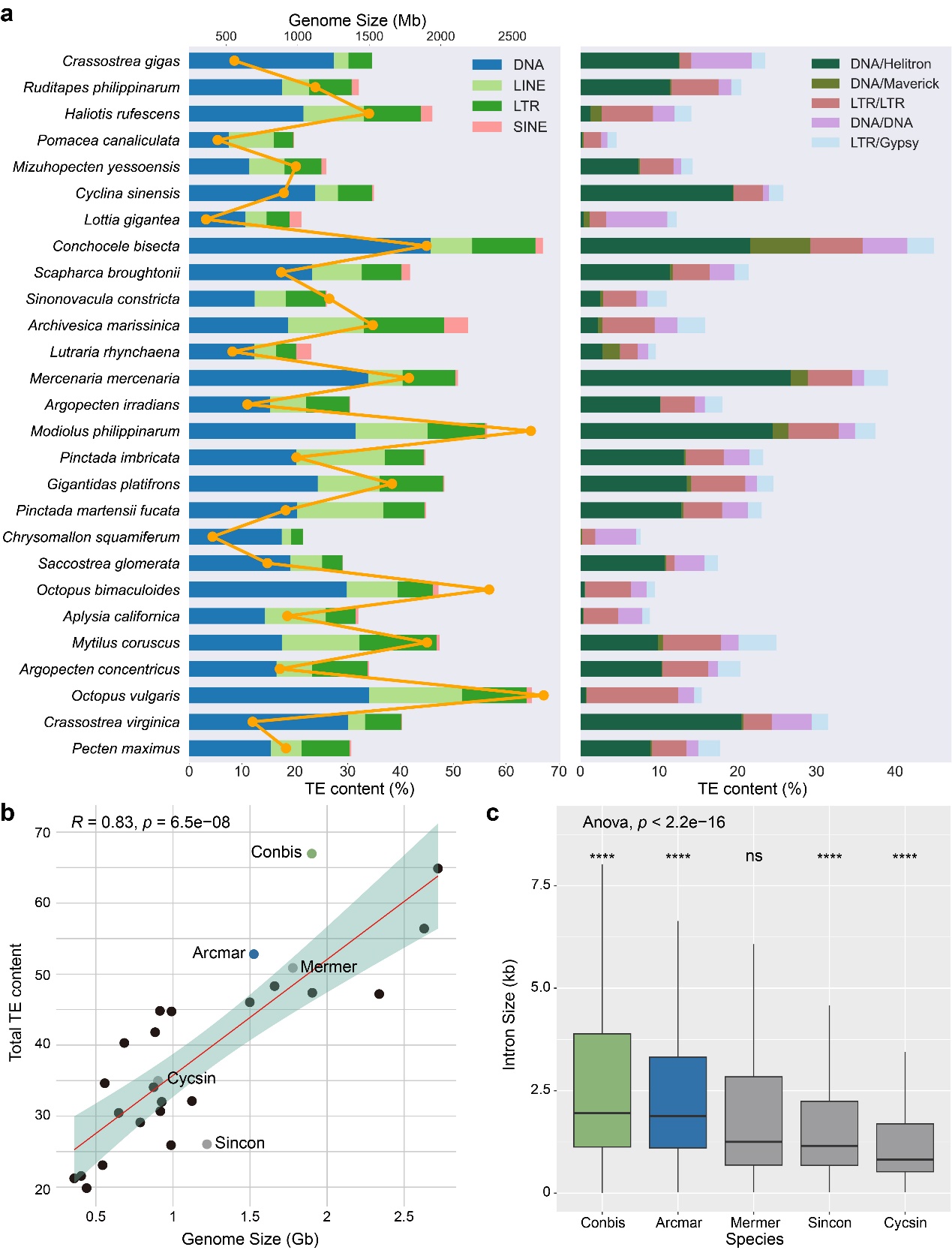


**Supplementary Figure 10** Relationship between genome size and transposable elements (TE) in the lophotrochozoan genomes. **(a)** Genome size (yellow dots linked by lines) and TE type and subtype content in 27 lophotrochozoan genomes are shown. **(b)** Correlation between Genome size and Total TE content among the 27 species. The red line represents the fitted linear regression line and the cyan bands represent the 95% confidence interval bands. Genomes of symbiotic *Conchocele bisecta* (Conbis), symbiotic *Archivesica marissinica* (Arcmar), and three asymbiotic Heteroconchia clams (*Cyclina sinensis* (Cycsin), *Sinonovacula constricta* (Sincon) *Mercenaria mercenaria* (Mermer)) were highlighted in green, blue, and grey, respectively. **(c)** Distribution of intron sizes in the five Heteroconchia clams. Asterisks above the box represents significant difference (*p* < 0.0001) when compared to *Mercenaria mercenaria* (Mermer) using T-test.

# Supplementary Figure 11


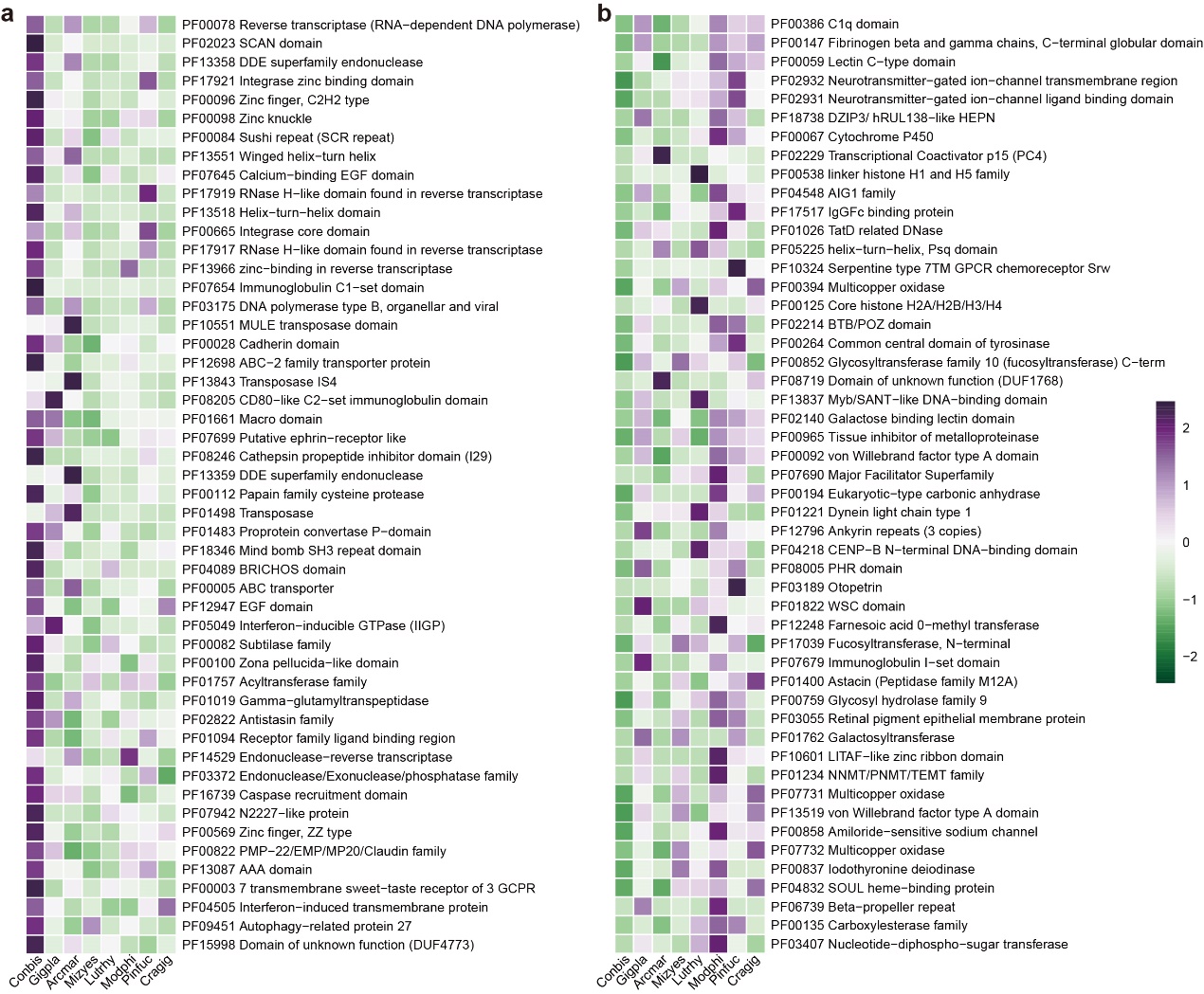


**Supplementary Figure 11** Expanded or contracted Pfam domains in *Conchocele bisecta*. Top 50 significant expanded (a) or contract (b) domains in *C. bisecta* (Conbis) are shown, when compared with other five asymbiotic bivalves (Lutrhy: *Lutraria rhynchaena*; Mizyes: *Mizuhopecten yessoensis*; Modphi: *Modiolus philippinarum*; Pinfuc: *Pinctada fucata*; Cragig: *Crassostrea gigas*), and the counts of domains in two endosymbiotic bivalves (Arcmar: *Archivesica marissinica*; Gigpla: *Gigantidas platifrons*) are also shown.

# Supplementary Figure 12


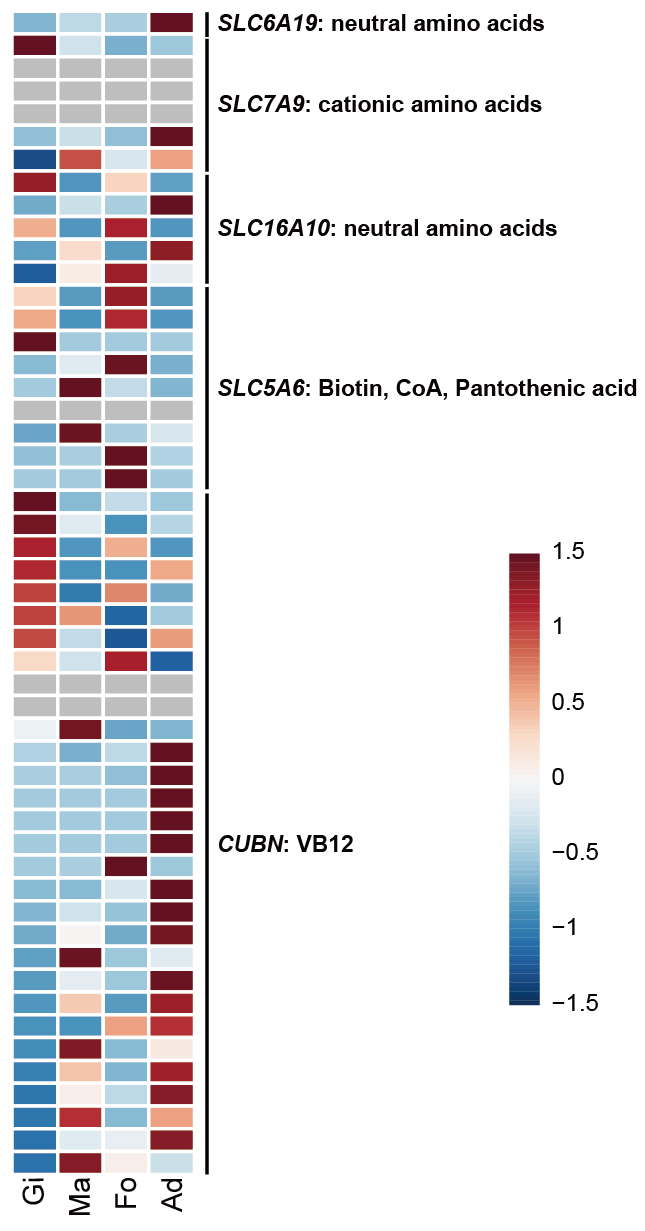


**Supplementary Figure 12** Expression levels of genes related in transport of rare metabolites (*Slc16a10*, *Slc5a6*, *cubilin*) in different tissues (Gi, gill; Ma, mantle; Fo, foot; Ad, adductor) of *Conchocele bisecta* based on transcriptomic data.

# Supplementary Figure 13


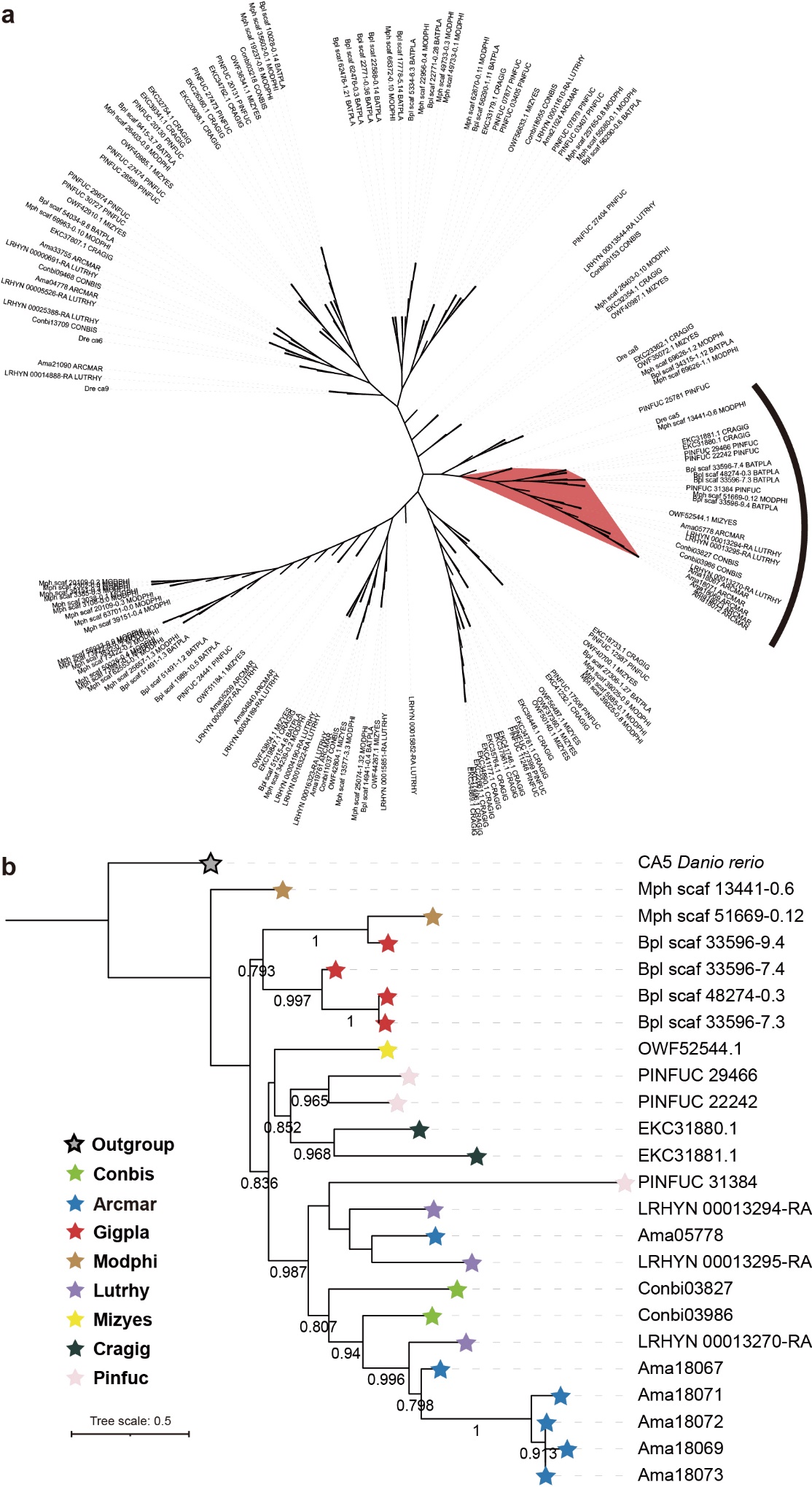


**Supplementary Figure 13** Carbonic anhydrases (CAs) in bivalves. a Phylogenetic tree of all the carbonic anhydrases in the 8 bivalves based on KEGG annotation. The clade with red background has been reported to undergo duplication in *Archivesica marissinica* and *Gigantidas platifrons*. b Phylogenetic tree of the carbonic anhydrases belong to the red-backgrounded clade (a) in the 8 bivalves (Conbis: *Conchocele bisecta*; Arcmar: *Archivesica marissinica*; Gigpla: *Gigantidas platifrons*; Lutrhy: *Lutraria rhynchaena*; Mizyes: *Mizuhopecten yessoensis*; Modphi: *Modiolus philippinarum*; Pinfuc: *Pinctada fucata*; Cragig: *Crassostrea gigas*; Outgroup: *Homo sapiens*).

# Supplementary Figure 14


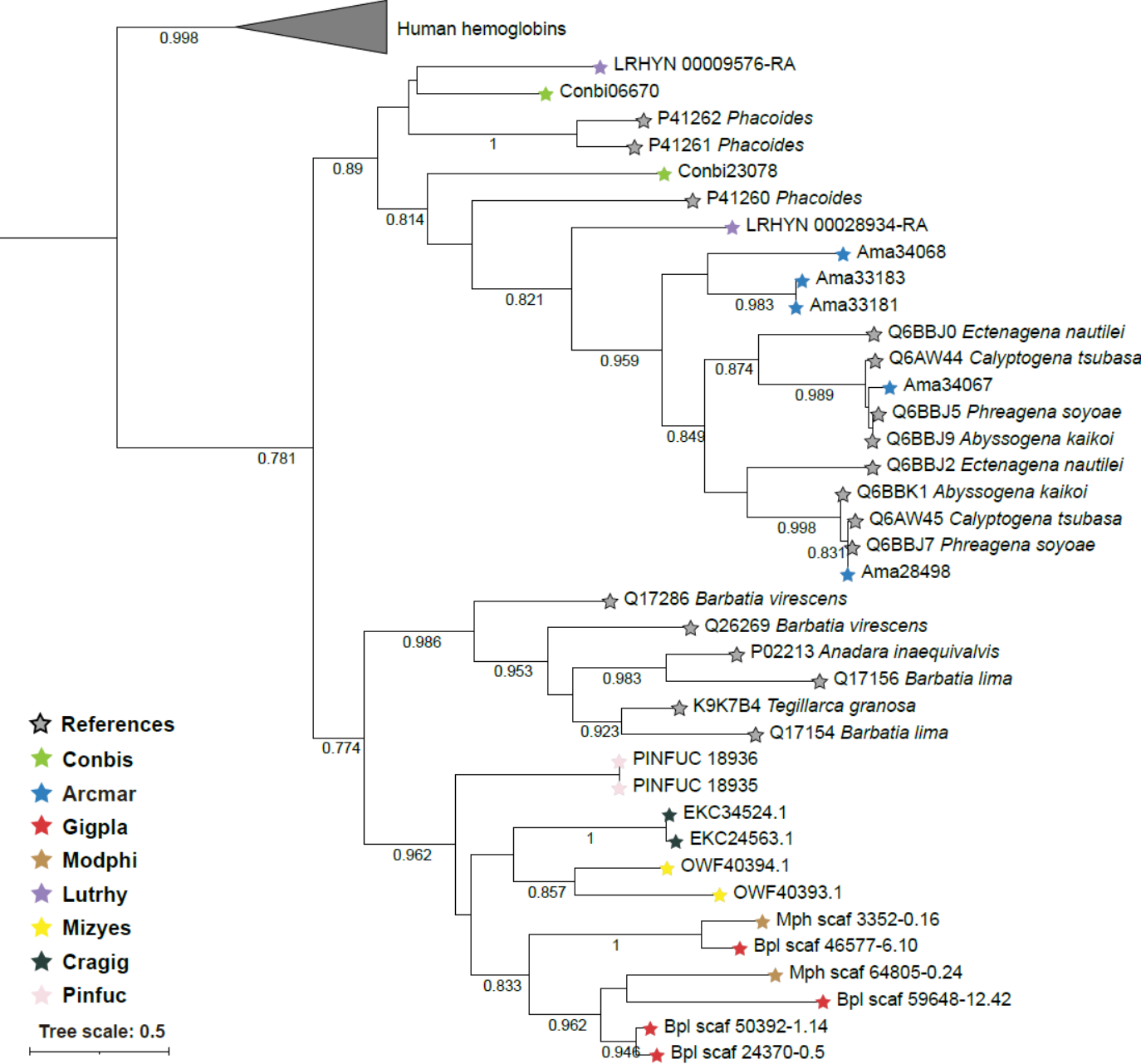


**Supplementary Figure 14** Hemoglobin and hemoglobin-like proteins (Hbs) in bivalves. The phylogenetic tree were revised from Ip et al. 2020, and shallow-water bivalve references were obtained from UniProt. There were four copies of Hbs in *Gigantidas platifrons* and five copies in *Archivesica marissinica*, while there were two copies in most of other bivalves (Conbis: *Conchocele bisecta*; Arcmar: *A. marissinica*; Gigpla: *G. platifrons*; Lutrhy: *Lutraria rhynchaena*; Mizyes: *Mizuhopecten yessoensis*; Modphi: *Modiolus philippinarum*; Pinfuc: *Pinctada fucata*; Cragig: *Crassostrea gigas*; Outgroup: *Homo sapiens*).

# Supplementary Figure 15


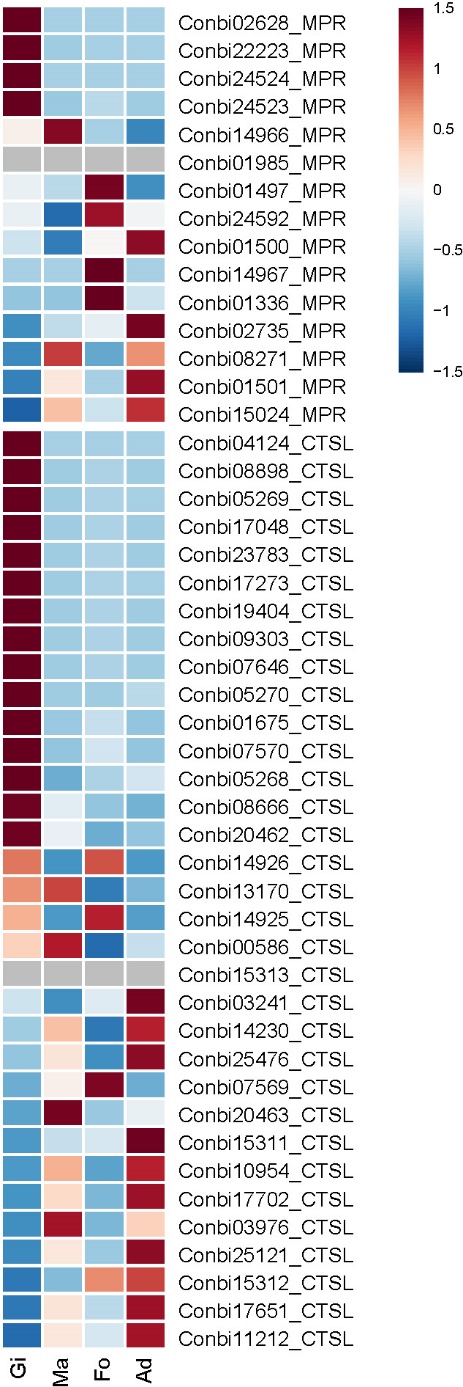


**Supplementary Figure 15** Expression levels of expanded genes that implicated in phagocytosis (*MPR* and *cstL*) in different tissues (Gi, gill; Ma, mantle; Fo, foot; Ad, adductor) of *Conchocele bisecta* based on transcriptomic data.

# Supplementary Figure 16


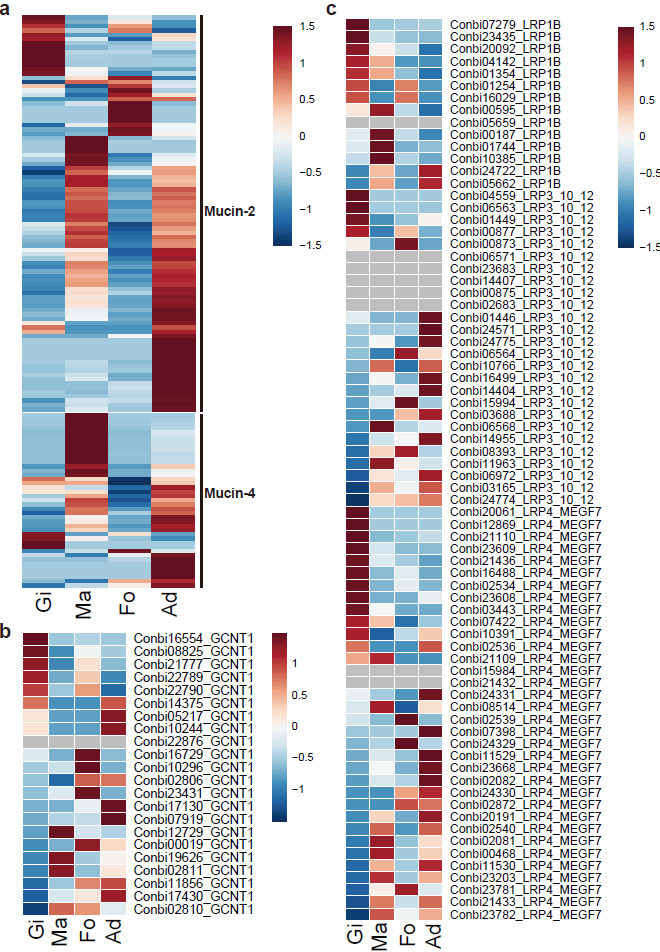


**Supplementary Figure 16** Expression levels of expanded genes that implicated in recognition and homeostasis, including mucins, gcnt1 and LRPs, in different tissues (Gi, gill; Ma, mantle; Fo, foot; Ad, adductor) of *Conchocele bisecta* based on transcriptomic data.

# Supplementary Table 2

**Table S2** Genomic statistics of the SCbi (Symbionts of *Conchocele biescta*)

| **Class** | **SCbi** |
| --- | --- |
| Genome Size (Mb) | 1.33 |
| Number of scaffolds | 45 |
| Contig N50 (kb) | 146.55 |
| Completeness (%) | 92.85 |
| Contamination rate (%) | 1.32 |
| Number of protein-coding genes | 1,095 |
| Number of 5S_rRNA | 1 |
| Length of 5S_rRNA (bp) | 107 |
| Number of 16S_rRNA | 1 |
| Length of 16S_rRNA (bp) | 1,521 |
| Number of 23S_rRNA | 1 |
| Length of 23S_rRNA (bp) | 2,911 |
| Number of tRNA | 31 |

# Supplementary Table 5

**Table S5** Distribution genes involved in amino acids biosynthesis in SUP05 bacteria (Presented (P) or missed in the genome)

| **Genome** | **NCBI Accession No.** | **Genome completeness (%)** | **Genes** | | | |
| --- | --- | --- | --- | --- | --- | --- |
|  |  |  | ***argH*** | ***lysA*** | ***leuB*** | ***hemC*** |
| **Symbiont of *Conchocele bisecta* (SCbi)** | This study | 92.85 | missed | missed | missed | missed |
| Symbiont of *Bathymodiolus azoricus* | GCA_001298715.2 | 97.68 | P | P | P | P |
| Symbiotic SOB in Haplosclerida sponge | GCA_008364125.1 | 96.02 | P | missed | missed | P |
| Symbiont of *Bathymodiolus septemdierum* | GCF_001547755.1 | 98.67 | P | P | P | P |
| Symbiont of *Bathymodiolus* spp. | GCA_900180385.1 | 98.01 | P | P | P | P |
| Symbiotic SOB in cold seep sponge | GCA_002007405.1 | 98.01 | P | P | P | P |
| *Candidatus Thioglobus pontius* UU116 | GCA_014384345.1 | 97.35 | P | P | P | P |
| *Candidatus Thioglobus autotrophicus* EF1 | GCF 001293165.1 | 99.17 | P | P | P | P |
| Gammaproteobacteria bacterium UBA8582 | GCA_003535415.1 | 82.79 | P | P | P | P |
| *Candidatus Ruthia* sp. UBA2013 | GCA_002335045.1 | 91.23 | P | P | P | P |
| Uncultured SUP05 cluster bacterium | GCA_000205985.2 | 85.52 | P | P | missed | P |
| *Candidatus Thioglobus singularis* PS1 | GCF_001281385.1 | 98.67 | P | P | P | P |

# Supplementary Table 6

**Table S6** Statistics of sequenced genomic data

| **Category** | **Bases number(bp)** | **genome coverage** | **read length** |
| --- | --- | --- | --- |
| PacBio Sequel II CLR reads Cell1 | 161,782,659,894 | 85.12 | Mean Length 19.48kb |
| PacBio Sequel II CLR reads Cell2 | 158,203,576,101 | 83.24 | Mean Length 17.88kb |
| BGI SEQ 500 short reads(clean) | 114,526,802,000 | 60.26 | PE100 |
| Hi-C | 396,226,549,120 | 208.47 | PE100 |

# Supplementary Table 7

**Table S7** Characteristics of the *Conchocele bisecta* genome assembly

| **Class** | **Values** |
| --- | --- |
| Total size (bp) | 1,900,675,896 |
| Number of contigs | 17,791 |
| Contig N50(bp) | 488,582 |
| Longest contig(bp) | 5,922,987 |
| Mean size(bp) | 106,834 |
| GC content(%) | 39.22 |
| Total length of retrotransposons (Class I, bp) | 463,387,700 |
| Total length of transposons (Class II, bp) | 918,942,640 |
| Total length of genes (bp) | 31,332,142 |
| Number of genes | 25,473 |
| Average gene length (bp) | 1,230 |
| Genome BUSCO | C:87.8%[S:85.1%,D:2.7%],F:4.7%,M:7.5% |
| Protein BUSCO | C:88.1%[S:85.1%,D:3.0%],F:7.3%,M:4.6% |

# Supplementary Table 8

**Table S8** The mapping rate of short reads to *Conchocele bisecta* genome

|  | **Total reads** | **Mapped reads** | **Mapped (%)** | **Properly mapped reads** | **Properly mapped (%)** |
| --- | --- | --- | --- | --- | --- |
| WGS short reads | 1,151,476,337 | 1,127,010,259 | 97.88 | 1,083,784,624 | 94.12 |

# Supplementary Table 9

**Table S9** Chromosome statistics of *Conchocele bisecta* genome

| **Pseudo-chromosome** | **Length** |
| --- | --- |
| chr1 | 225,641,443 |
| chr2 | 160,689,902 |
| chr3 | 137,241,376 |
| chr4 | 126,956,079 |
| chr5 | 122,994,810 |
| chr6 | 109,599,820 |
| chr7 | 106,883,709 |
| chr8 | 98,840,267 |
| chr9 | 98,770,060 |
| chr10 | 98,395,754 |
| chr11 | 94,468,294 |
| chr12 | 88,168,022 |
| chr13 | 85,866,967 |
| chr14 | 80,201,555 |
| chr15 | 75,987,425 |
| chr16 | 71,990,154 |
| chr17 | 64,664,772 |

# Supplementary Table 10

**Table S10** TE contents (%) of lophotrochozoan genomes

| **Species** | **TE content** | **Retro^b^** | **LINE** | **LTR** | **SINE** | **DNA^c^** |
| --- | --- | --- | --- | --- | --- | --- |
| ***Conchocele bisecta* (S)^a^** | 66.96 | 21.23 | 45.72 | 7.85 | 11.97 | 1.42 |
| *Octopus vulgaris* | 64.87 | 30.79 | 34.08 | 17.58 | 12.21 | 1.01 |
| ***Modiolus philippinarum*** | 56.41 | 24.9 | 31.5 | 13.66 | 10.85 | 0.39 |
| ***Archivesica marissinica* (S)** | 52.81 | 34.07 | 18.73 | 14.31 | 15.22 | 4.54 |
| ***Mercenaria mercenaria*** | 50.87 | 17.01 | 33.87 | 6.53 | 10.01 | 0.46 |
| ***Gigantidas platifrons* (S)** | 48.31 | 23.96 | 24.35 | 11.67 | 12.06 | 0.23 |
| ***Mytilus coruscus*** | 47.37 | 29.81 | 17.56 | 14.68 | 14.56 | 0.57 |
| *Octopus bimaculoides* | 47.19 | 17.33 | 29.86 | 9.6 | 6.64 | 1.08 |
| *Haliotis rufescens* | 46.03 | 24.41 | 21.63 | 11.49 | 10.71 | 2.21 |
| ***Pinctada martensii fucata*** | 44.82 | 24.38 | 20.45 | 16.27 | 7.82 | 0.28 |
| ***Pinctada imbricata*** | 44.74 | 24.44 | 20.3 | 16.7 | 7.44 | 0.3 |
| ***Scapharca broughtonii*** | 41.81 | 18.55 | 23.26 | 9.44 | 7.53 | 1.58 |
| ***Crassostrea virginica*** | 40.3 | 10.23 | 30.08 | 3.22 | 6.9 | 0.11 |
| ***Cyclina sinensis*** | 34.96 | 11.07 | 23.89 | 4.29 | 6.45 | 0.34 |
| ***Crassostrea gigas*** | 34.63 | 7.24 | 27.39 | 2.76 | 4.45 | 0.03 |
| ***Argopecten concentricus*** | 34.07 | 17.54 | 16.53 | 6.74 | 10.5 | 0.29 |
| ***Ruditapes philippinarum*** | 32.12 | 14.55 | 17.57 | 5.17 | 8.02 | 1.36 |
| *Aplysia californica* | 32.01 | 17.65 | 14.36 | 11.51 | 5.62 | 0.52 |
| ***Pecten maximus*** | 30.69 | 15.21 | 15.48 | 5.79 | 9.08 | 0.34 |
| ***Argopecten irradians*** | 30.45 | 15.11 | 15.34 | 6.8 | 8.17 | 0.14 |
| ***Saccostrea glomerata*** | 29.11 | 9.96 | 19.15 | 6.03 | 3.86 | 0.07 |
| ***Sinonovacula constricta*** | 26.04 | 13.63 | 12.41 | 5.89 | 7.53 | 0.21 |
| ***Mizuhopecten yessoensis*** | 25.91 | 14.53 | 11.38 | 6.68 | 6.98 | 0.87 |
| ***Lutraria rhynchaena*** | 23.09 | 10.73 | 12.36 | 4.11 | 3.78 | 2.83 |
| *Chrysomallon squamiferum* | 21.57 | 4.04 | 17.53 | 1.72 | 2.28 | 0.04 |
| *Lottia gigantea* | 21.23 | 10.56 | 10.67 | 3.99 | 4.32 | 2.24 |
| *Pomacea canaliculata* | 19.86 | 12.37 | 7.49 | 8.55 | 3.73 | 0.09 |

^a^Bolded species are bivalves, and “S” indicates symbiotic bivalves

^b^Retro: Retrotransposons

^c^DNA: DNA transposons
